# Targeting asparagine potentiates anti-PD-L1 immunotherapy in gastric cancer by enhancing CD8^+^ T cell anti-tumor response

**DOI:** 10.3389/fimmu.2025.1626581

**Published:** 2025-10-28

**Authors:** Mingpai Ge, Longfei Wang, Bowen Zheng, Lu Zhan, Lu Cui, Han Wang, Enyuan Huang, Yuan Xu, Xusheng Chang, Zhaorui Liu, Jun Xu, Kai Yin

**Affiliations:** ^1^ Department of Gastrointestinal Surgery, Changhai Hospital, Naval Medical University, Shanghai, China; ^2^ Ruijin Hospital Affiliated with Shanghai Jiao Tong University School of Medicine, Shanghai, China; ^3^ Shi Dong Hospital, Shanghai, China; ^4^ School of Pharmacy Shanghai Engineering Research Center of Immunotherapeutics, Fudan University, Shanghai, China; ^5^ Department of Pharmacy, The First Hospital of Putian City, Putian, Fujian, China; ^6^ Tianjin Medical University General Hospital, Tianjin, China; ^7^ Department of Internal Medicine, Zhongnan Hospital of Wuhan University, Wuhan University, Wuhan, China

**Keywords:** gastric cancer, metabolism, immunotherapy, TME (tumor microenvironment), CD8 T cell, asparagine

## Abstract

**Introduction:**

Immunotherapy efficacy in gastric cancer (GC) is often constrained by the tumor microenvironment (TME), which is profoundly influenced by aberrant metabolism. Asparagine, an amino acid critical for neoplastic proliferation, also modulates CD8+ T cell metabolic programming. We investigated the impact of targeting asparagine on the GC immune microenvironment and its potential to synergize with anti-PD-L1 therapy.

**Methods:**

The therapeutic efficacy of asparagine targeting was evaluated in GC tumor models. CD8+ T cell populations within the TME were analyzed by flow cytometry, while cytokine and chemokine levels (IFN-γ, GZMB, CXCL9, CXCL10) were quantified by ELISA. The effects on CD8+ T cell activation and antitumor function were assessed in vitro and in vivo. Synergistic efficacy with anti-PD-L1 therapy was evaluated in GC models, and the dependency on CD8+ T cells was confirmed via antibody-mediated depletion experiments.

**Results:**

Targeting asparagine inhibited GC growth in vitro and in vivo, implicating immune system involvement. Mechanistically, asparagine targeting significantly increased the proportion of CD8+ T cells within the TME and upregulated the expression of IFN-γ, GZMB, CXCL9, and CXCL10. Furthermore, combining asparagine targeting with anti-PD-L1 therapy produced synergistic antitumor activity. This combined therapeutic effect was significantly attenuated by the depletion of CD8+ T cells.

**Discussion:**

Our findings indicate that targeting asparagine promotes CD8+ T cell activation and infiltration, thereby remodeling the GC immune microenvironment to enhance host antitumor immunity. The combination of asparagine targeting with anti-PD-L1 therapy elicits potent, synergistic antitumor effects that are demonstrably dependent on CD8+ T cells. This study provides a strong rationale for targeting asparagine metabolism as a novel strategy to improve immunotherapeutic outcomes in GC.

## 1 Introduction

Gastric cancer (GC) stands as a major global malignancy, ranking as the fifth most prevalent cancer and the fourth leading cause of cancer-related mortality worldwide (1). While conventional therapeutic modalities, including surgery, chemotherapy, and radiotherapy, have contributed to improved patient survival, their long-term efficacy remains suboptimal, and their associated substantial toxicities significantly compromise patients’ quality of life (2–4). In recent years, tumor immunotherapy has emerged as a transformative therapeutic strategy, revolutionizing the field of cancer treatment (5, 6). Nevertheless, the inherent complexity of the GC immune microenvironment imposes significant limitations on the effectiveness of immunotherapy, consequently restricting the clinical benefit for a considerable proportion of GC patients (7–11).

The reciprocal regulation between tumor metabolism and the immune microenvironment has emerged as a central theme in contemporary cancer research. Tumor cell metabolic reprogramming not only fulfills the bioenergetic and biosynthetic demands underpinning their proliferation and survival but also critically shapes the cellular and molecular milieu of the TME and dictates the functional phenotype of resident immune cells (12–16). This metabolic rewiring confers distinct “metabolic vulnerabilities” upon neoplastic cells, differentiating them from their normal counterparts through altered patterns of nutrient acquisition and metabolite efflux, thus providing a compelling rationale for the development of novel therapeutic strategies predicated on targeting tumor metabolism (17–21). Intriguingly, asparagine metabolism is recognized to modulate immune cell function within the TME, particularly impacting CD8^+^ T lymphocytes. CD8^+^ T lymphocytes are widely acknowledged as pivotal mediators of effective anti-tumor immunity, with their infiltrating density and functional competence within the TME being critical determinants of the potency of the host anti-tumor response (22–24). Asparaginase is a clinically validated therapeutic agent employed in the management of leukemia, exhibits a favorable and well-characterized safety profile (25–28). Notwithstanding substantial progress in the clinical management of GC on a global scale, the potential therapeutic synergy of targeting asparagine in conjunction with immunotherapy warrants further investigation and remains relatively underexplored. Collectively, considering its capacity to exert a dual influence on both gastric carcinoma cell proliferation and resident immune cell populations within the TME, targeting asparagine holds considerable promise as a therapeutic strategy for augmenting immunotherapeutic outcomes in GC.

In this study demonstrates that targeting asparagine inhibits the proliferation of GC cells and promotes both the proliferative activity and antitumor efficacy of CD8^+^ T cells. Furthermore, it effectively suppresses GC growth and significantly enhances CD8^+^ T cell infiltration within the GC immune microenvironment *in vivo*. These findings collectively provide a theoretical rationale for combining asparagine targeting with immunotherapy. Specifically, the combination of targeting asparagine and anti-PD-L1 therapy can exert significant anti-tumor effects *in vivo*, and the efficacy of this combined treatment is dependent on CD8^+^ T cell infiltration within the tumor immune microenvironment. In summary, targeting asparagine not only suppresses GC cell proliferation but also reshapes the GC immune microenvironment by improving CD8^+^ T cell infiltration, thereby augmenting the efficacy of immunotherapy for GC.

## 2 Results

### 2.1 Targeting asparagine leads to the inhibition of MFC proliferation

**Figure 1:**
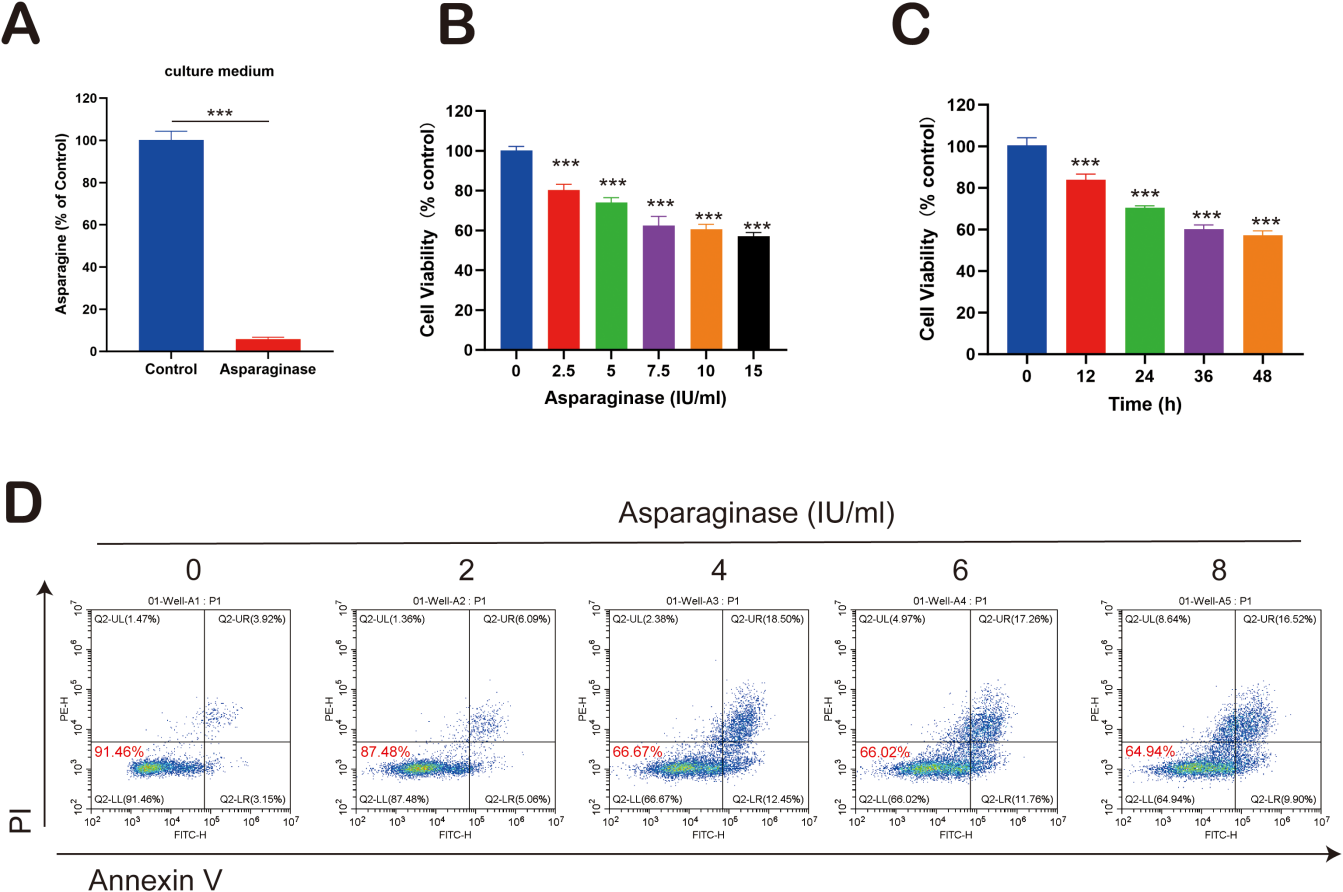
**(A)** Asparagine concentration in the cell culture medium. **(B, C)** MFC cell viability changes following asparaginase treatment for various asparaginase concentrations and durations. **(D)** Apoptosis in MFC cells under treatment with varying asparaginase concentrations. *** indicates p-value < 0.001.

Asparaginase was employed as an asparagine-targeting therapeutic agent. First, its capacity to degrade asparagine in culture medium was assessed. Cell culture medium was treated with asparaginase at 4 IU/ml for 24 hours. Compared to the control group, asparaginase significantly degraded asparagine in the medium (**Figure 1A**). Subsequently, the *in vitro* effect of asparaginase on the viability of MFC tumor cells was determined using the CCK-8 assay after treatment for 24 hours with varying concentrations (0, 1, 2, 4, 6, 8 IU/ml). Asparaginase significantly inhibited the viability of MFC cells, and this inhibitory effect increased significantly with rising asparaginase concentration (**Figure 1B**). Furthermore, the time-dependent effect of asparaginase (4 IU/ml) on MFC cell viability was evaluated using the CCK-8 assay at different time points (0, 12, 24, 36, 48 hours). Asparaginase significantly inhibited MFC cell viability, and cell viability significantly decreased with increasing asparaginase incubation time (**Figure 1C**). Collectively, these results demonstrated that the inhibitory effect of asparaginase on MFC cell proliferation is both concentration-dependent and time-dependent. To assess the extent of cell apoptosis, Annexin V-PI staining followed by flow cytometry was performed. Annexin V-PI stains necrotic and apoptotic cells, while viable cells remain unstained. MFC cells were treated *in vitro* with varying concentrations of asparaginase (0, 2, 4, 6, 8 IU/ml) for 24 hours before flow cytometry analysis. The percentage of viable cells significantly decreased with increasing asparaginase concentration, while the percentage of necrotic and apoptotic cells significantly increased with rising asparaginase concentration (**Figure 1D**). In summary, these findings indicate that asparagine deprivation induced by asparaginase can inhibit MFC cell proliferation.

To elucidate the molecular mechanism by which Asparaginase inhibits the viability of MFC cells, we first assessed the impact of exogenous asparagine supplementation. Treatment with 4 IU/ml Asparaginase for 48 hours markedly suppressed MFC cell viability compared to the control group. To confirm that this effect was attributable to asparagine depletion, we co-treated cells with Asparaginase and exogenous asparagine (ASN). This co-treatment significantly rescued the Asparaginase-induced loss of cell viability (**Supplementary Figure S1A**). Notably, treatment with ASN alone enhanced cell proliferation, suggesting that asparagine is a critical nutrient for MFC cells.

Subsequently, we investigated the underlying molecular changes via Western blot. Asparaginase treatment led to a significant upregulation of Asparagine Synthetase (ASNS), indicating the activation of a compensatory mechanism in response to asparagine starvation. This upregulation was reversed to near-control levels upon ASN supplementation (**Supplementary Figure S1B**). Given that autophagy is a critical pro-survival response to nutrient deprivation, we also examined its status. Asparaginase treatment significantly increased the levels of the autophagic marker LC3-II. Consistent with our other findings, this induction of autophagy was effectively reversed by ASN supplementation (**Supplementary Figure S1C**).

Taken together, these results demonstrate that Asparaginase exerts its cytotoxic effects by depleting extracellular asparagine, which in turn triggers a compensatory upregulation of ASNS and induces protective autophagy in MFC cells. These effects are reversible upon the restoration of exogenous asparagine.

### 2.2 Targeting asparagine enhances CD8+ T cell infiltration in the gastric TME

**Figure 2:**
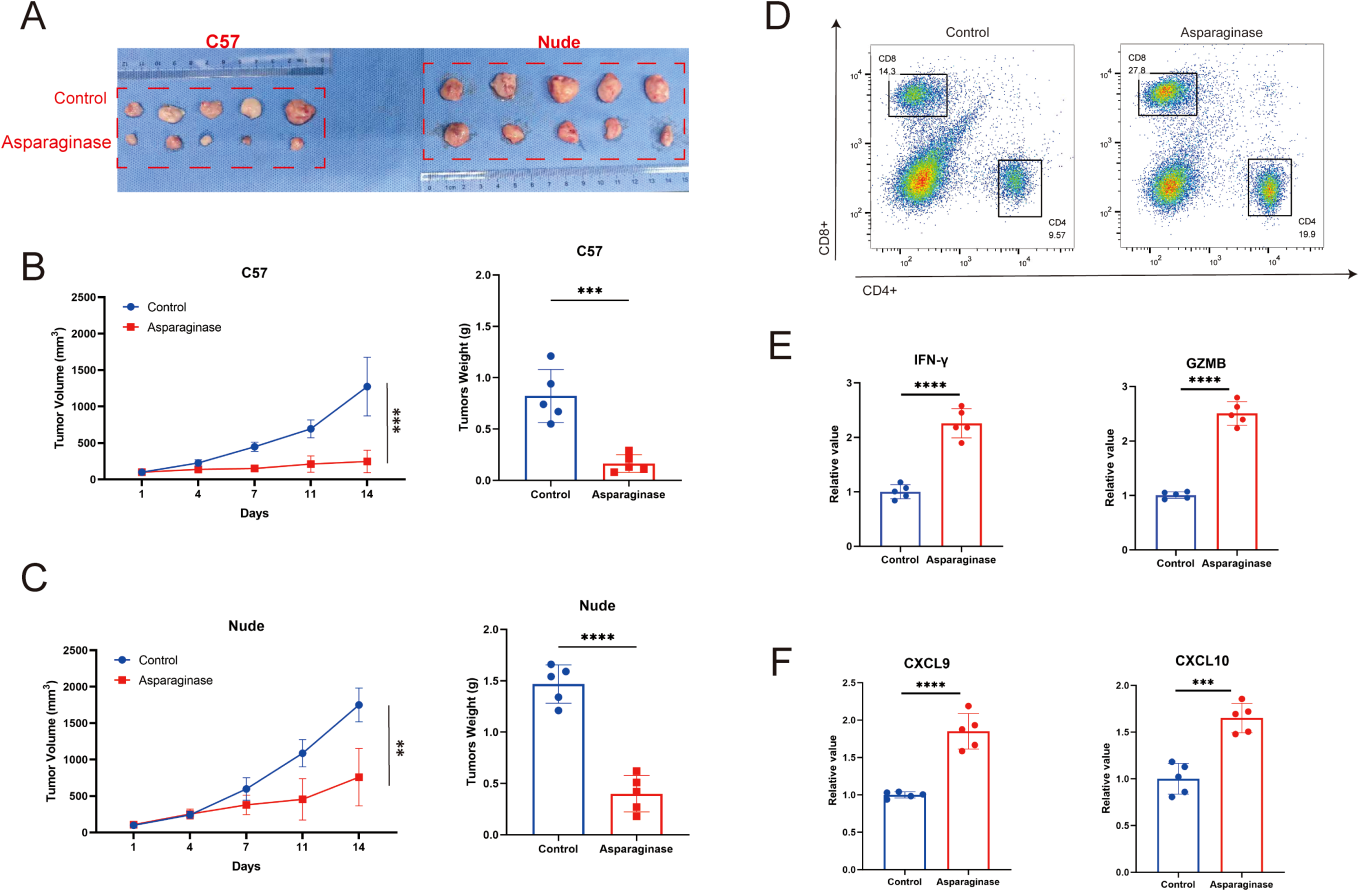
**(A–C)** The *in vivo* antitumor effect of asparaginase was investigated in an MFC tumor model (n=5 mice per group). Tumor volume was assessed twice weekly, and treatment was initiated when the tumor volume reached approximately 100 mm (3). **(D)** The proportion of CD4/CD8 positive cells in the tumor tissue in each group after asparaginase treatment. **(E, F)** IFN-γ, GZMB, CXCL9 and CXCL10 in tumors were measured by ELISA after asparaginase treatment (n = 5). ** indicates p-value < 0.01, *** indicates p-value < 0.001 and **** indicates p-value < 0.0001.

Subcutaneous MFC tumor-bearing models were established in both C57 mice and nude mice to evaluate the *in vivo* anti-tumor effect of targeting asparagine. As shown in **Figures 2A–C**, after 14 days of asparagine-targeting treatment, both the volume and weight of the tumors were significantly reduced. Notably, comparing the post-treatment tumor volumes revealed that the anti-tumor effect of asparagine-targeting treatment was more pronounced in C57 mice than in nude mice. Given the deficiency in mature T cells in nude mice, it is hypothesized that T cell-mediated immune mechanisms may contribute to the therapeutic process of targeting asparagine in GC. To investigate the role of immune cells, flow cytometry was performed on tumors harvested from C57 mice to analyze the immune cell composition within the TME. Compared to the control group, the proportion of CD8^+^ T cells in the TME were markedly increased in the asparaginase-treated group (**Figure 2D**). This finding suggests that targeting asparagine enhances CD8^+^ T cell infiltration into the TME. Furthermore, the levels of the key immune effectors IFN-γ and Granzyme B (GZMB) were measured in the GC tissue. Both IFN-γ and GZMB levels were significantly higher in the asparaginase group compared to the control group (**Figure 2E**), indicating that targeting asparagine promotes the activation of immune cells within the TME. The levels of the T cell chemoattractants CXCL9 and CXCL10 were also assessed in the GC tissue of both groups. The levels of CXCL9 and CXCL10 in the TME following asparagine-targeting treatment were significantly higher than those in the control group (**Figure 2F**). Collectively, these results demonstrate that targeting asparagine improves CD8^+^ T cell infiltration and enhances immune activation within the TME, which may contribute to its anti-tumor efficacy.

### 2.3 Targeting asparagine promotes CD8^+^ T cell proliferation and immune responses

**Figure 3:**
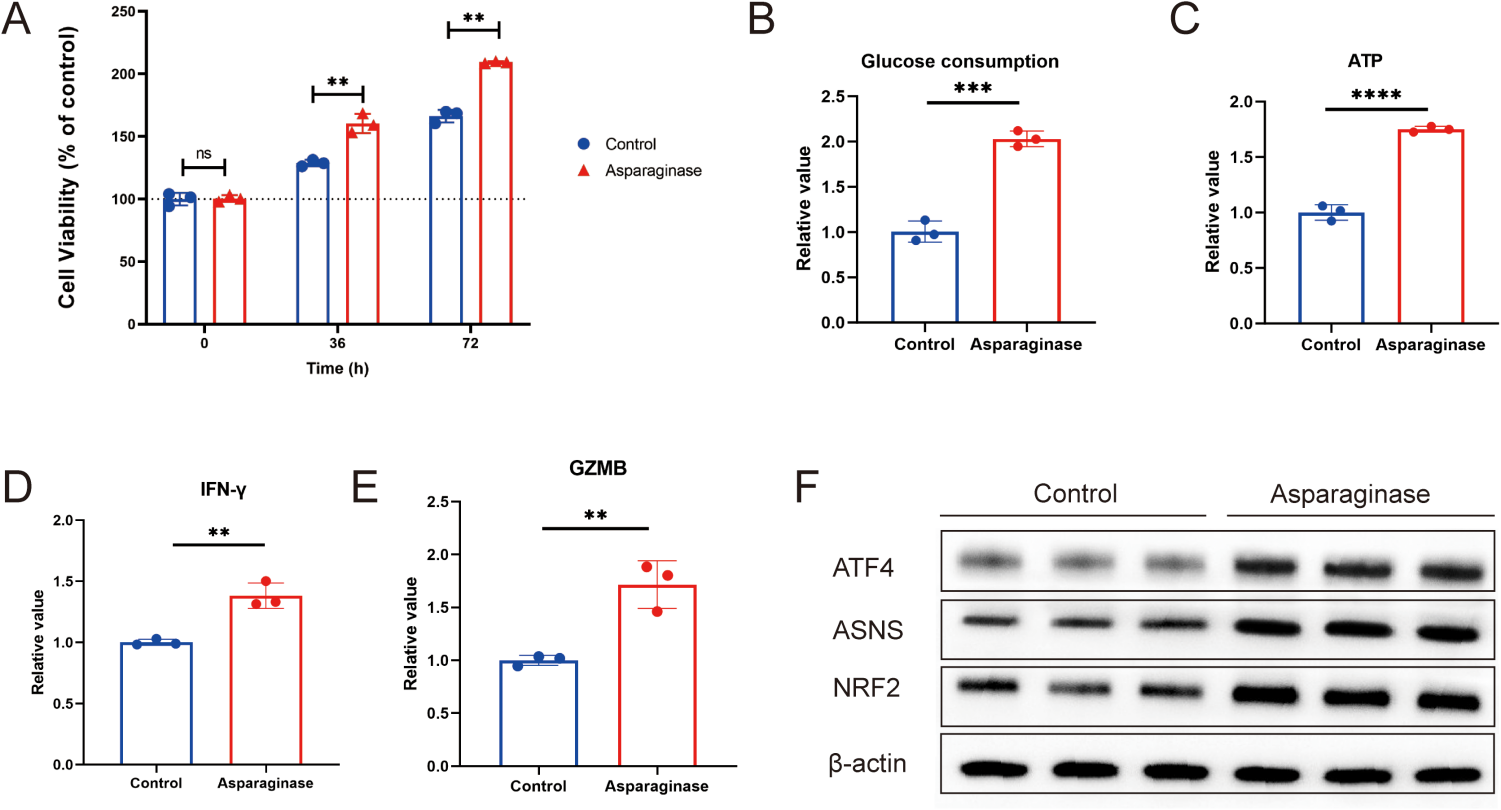
**(A)** Cell viability of CD8+ T cells activated with asparaginase, assessed at 36 h and 72 (h) **(B)** Glucose consumption, determined by the change in glucose concentration in the culture medium. **(C)** ATP level was determined by calculating the difference in CD8+T cells. **(D, E)** IFN-γ and GZMB in the supernatant from co-cultures of MFC and CD8+ T cells were measured by ELISA after asparaginase treatment. **(F)** Western blot analysis of the effect of asparaginase on the protein expression levels of ATF4, NRF2, and ASNS in CD8+T cells. ** indicates p-value < 0.01, ns means no significance.

To investigate the effects of asparagine deprivation on the proliferation and effector function of mouse CD8^+^ T cells, *in vitro* experiments were conducted using isolated mouse CD8^+^ T cells. CD8^+^ T cells were extracted from mouse spleens and cultured *in vitro*. After stabilization, cells were treated with 4 IU/ml asparaginase for 0, 36, and 72 hours to assess changes in cell viability. Asparaginase significantly promoted the proliferation of mouse CD8^+^ T cells (**Figure 3A**). Cell proliferation requires substantial glucose consumption and ATP production. To further evaluate the impact of asparagine deprivation on CD8^+^ T cell proliferative function, we measured glucose consumption and ATP levels. Asparagine deprivation significantly increased glucose consumption and elevated intracellular ATP content (**Figures 3B, C**), consistent with enhanced proliferation. To assess the effector function of mouse CD8^+^ T cells under asparagine deprivation, CD8^+^ T cells were co-cultured with MFC cells and treated with 4 IU/ml asparaginase for 48 hours. We measured the production of key effector cytokines, IFN-γ and GZMB. Asparaginase treatment significantly promoted the production of both IFN-γ and GZMB by activated CD8^+^ T cells (**Figures 3D, E**), indicating that asparagine deprivation significantly enhances CD8^+^ T cell effector function.

To investigate the underlying molecular mechanisms, we considered that nutrient deprivation activates the metabolism reprogram, which promotes cellular adaptation and enhances survival. A key molecule in the metabolism is ATF4, which can regulate downstream targets such as ASNS and NRF2 (29–32). ASNS is an enzyme involved in intracellular asparagine metabolism (21, 33–37), while NRF2 is a crucial regulator of intracellular carbon metabolism (30, 38–41). We used Western blot analysis to examine the effect of asparagine deprivation on the ATF4-NRF2-ASNS pathway in CD8^+^ T cells. We found that the expression levels of ATF4, NRF2, and ASNS were all significantly upregulated following asparagine deprivation (**Figure 3F**). Collectively, these results demonstrate that targeting asparagine promotes CD8^+^ T cell proliferation and immune response, and this effect may be associated with metabolic reprogramming regulated by the ATF4-NRF2-ASNS pathway.

### 2.4 Targeting asparagine and anti-PD-L1 therapy exhibit synergistic anti-tumor activity

**Figure 4:**
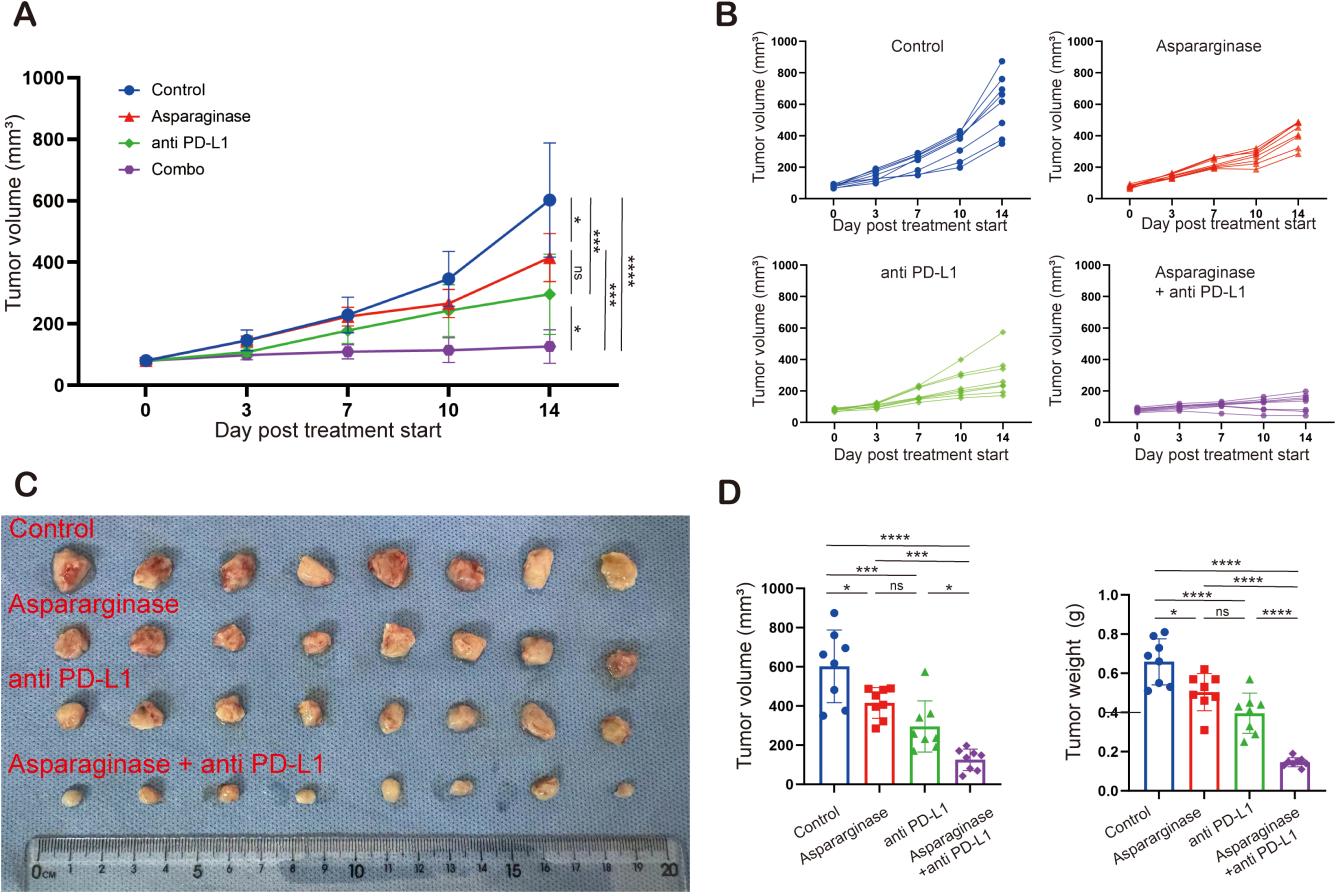
Targeting asparagine and PD-L1 elicited synergistic antitumor effect in gastric cancer. **(A–C)** The subcutaneous MFC tumor models (n= 8) were well-established in C57 mice. Tumor volume was measured twice a week. Treatment was initiated when the tumor volume reached 100 mm (3). **(D)** Statistical analysis of tumor volume and tumor weight. * indicates p-value < 0.05, ** indicates p-value < 0.01, *** indicates p-value < 0.001 and **** indicates p-value < 0.0001. ns means no significance.

To investigate the feasibility of targeting asparagine and PD-L1 in gastric tumor, C57 mice implanted with MFC tumors were treated with asparaginase, anti-PD-L1, and combination therapy. Compared to the control group, all three treatment groups significantly inhibited tumor growth. Tumor growth was most significantly inhibited in the combination therapy group compared to the control (**Figures 4A–C**). Furthermore, the average tumor volume in the combination therapy group was significantly reduced compared to either the asparaginase monotherapy group or the anti-PD-L1 monotherapy group. These results indicate that both asparaginase and anti-PD-L1 significantly slowed tumor growth as monotherapies, and targeting asparagine markedly enhanced the anti-tumor effect of anti-PD-L1. Assessment of tumor weight at the end of the treatment period showed average tumor weights control group were 658.75 ± 109.94 mg, asparaginase group were 500.00 ± 84.41 mg, anti-PD-L1 group were 396.25 ± 95.78 mg, and combination therapy group were 167.51 ± 21.65 mg. Compared to the control group, the asparaginase, anti-PD-L1, and combination therapy groups all significantly inhibited tumor growth, with the most pronounced therapeutic effect observed in the combination therapy group (**Figure 4D**).

**Figure 5:**
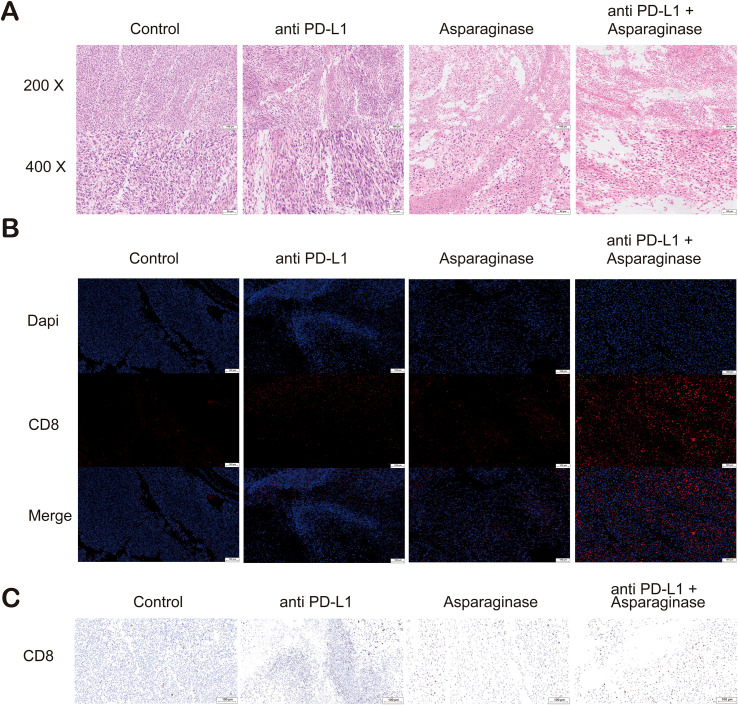
**(A)** Hematoxylin and eosin (H&E) staining of tumor sections. Compared with the control and anti-PD-L1 groups, extensive necrotic areas were observed in the tumor tissues from the asparaginase and combination therapy groups. **(B)** Immunofluorescence (IF) staining of tumor sections for CD8 (red) and nuclei (DAPI, blue). A significant increase in the infiltration of CD8^+^ T cells was observed in the tumor microenvironment of the asparaginase and combination therapy groups. **(C)** Immunohistochemical (IHC) staining for CD8 (brown) in tumor sections. Consistent with the immunofluorescence results, IHC analysis confirmed that asparaginase treatment, especially in combination with anti-PD-L1, dramatically increased the infiltration of CD8^+^ T cells into the tumors.

Pathological analysis of tumor tissues was performed. Tumor tissues from different groups were sectioned and stained with Hematoxylin and Eosin (H&E). Compared to the control group, tumor necrosis was significantly increased in the other three treatment groups. Tumor necrosis was most pronounced in the combination treatment group, which also presented with areas of cavitation (**Figure 5A**). CD8 immunohistochemistry (IHC) and immunofluorescence (IF) analysis were conducted on tumor tissues. In the fields of view of IF sections stained for CD8, the proportion of CD8^+^ cells, indicated by red immunofluorescence signal, was significantly increased in the three treatment groups compared to the control group; the immunofluorescence signal was strongest in the combination treatment group (**Figure 5B**). Similarly, in the fields of view of IHC sections stained for CD8, CD8-positive cells (indicated by brown staining) were significantly increased in all three treatment groups compared to the control group, with the highest percentage of CD8-positive cells observed in the combination treatment group (**Figure 5C**).

Collectively, these results indicate that combining anti-PD-L1 antibody with asparaginase significantly enhances the *in vivo* anti-tumor effect. This enhanced efficacy is likely associated with increased CD8^+^ T cell infiltration and improvement of the GC tumor immune microenvironment.

**Figure 6:**
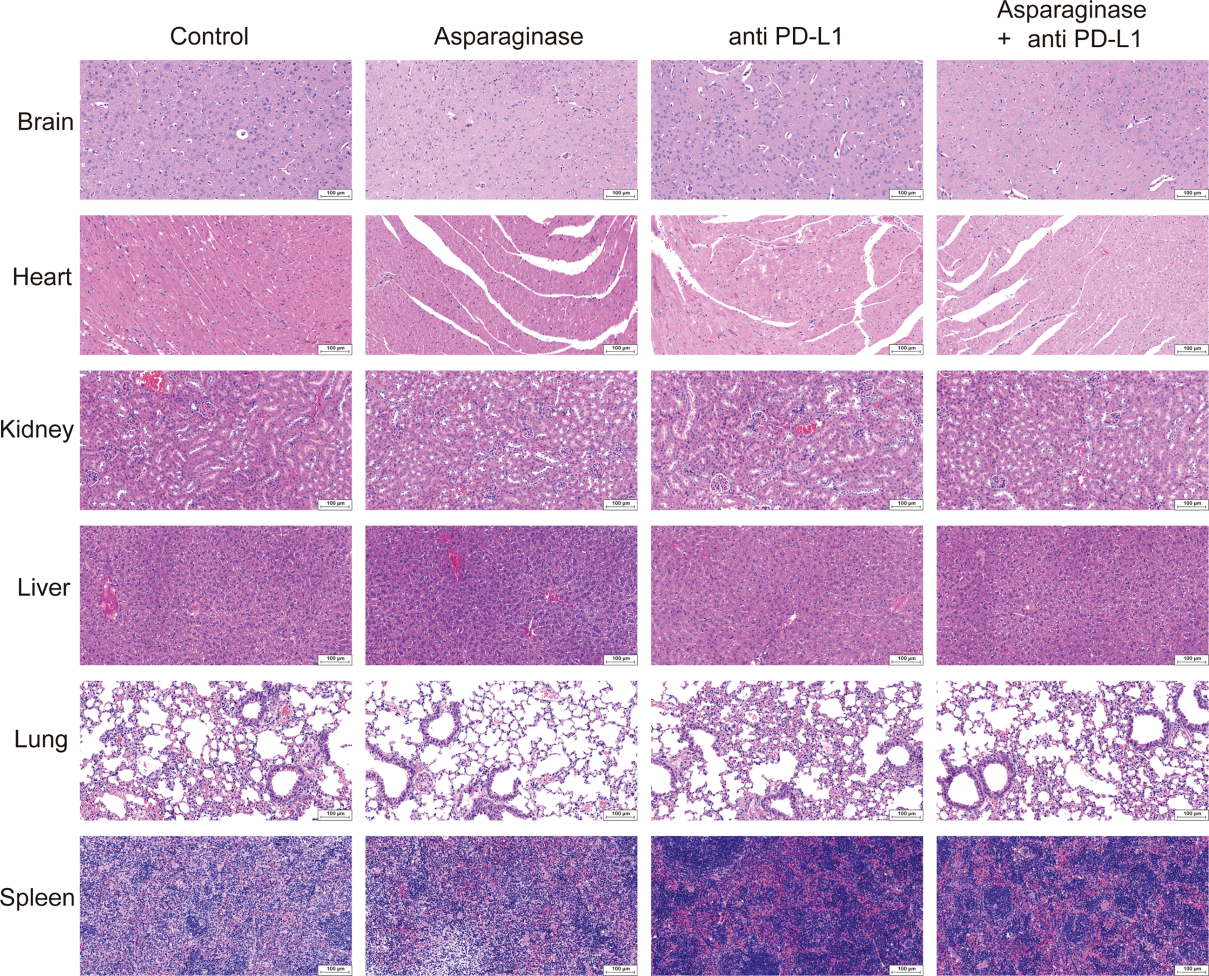
Hematoxylin and eosin staining of biosafety assessment.

Histological analysis by Hematoxylin and Eosin (H&E) staining was performed on the brain, heart, kidney, liver, lung, and spleen of mice to evaluate the potential organ toxicity of asparagine-targeting and anti-PD-L1 treatments. Compared to the control group, no remarkable pathological changes indicative of toxicity were observed in the organs of mice from the three treatment groups (**Figure 6A**).

### 2.5 The antitumor efficacy of the combined therapy was dependent on CD8+ T cells

To further validate the crucial role of CD8^+^ T cells in the anti-tumor effect of the asparagine-targeting and anti-PD-L1 combination therapy, CD8^+^ T cells and CD4^+^ T cells were specifically depleted *in vivo* using anti-mouse CD8 and anti-mouse CD4, respectively. These experiments aimed to determine whether the anti-tumor efficacy of the combination therapy is dependent on CD8^+^ T cells. MFC tumor-bearing mice models were established, and mice were assigned to the following treatment groups: Control, Asparaginase + anti-PD-L1, Asparaginase + anti-PD-L1 + anti-CD4, and Asparaginase + anti-PD-L1 + anti-CD8.

**Figure 7:**
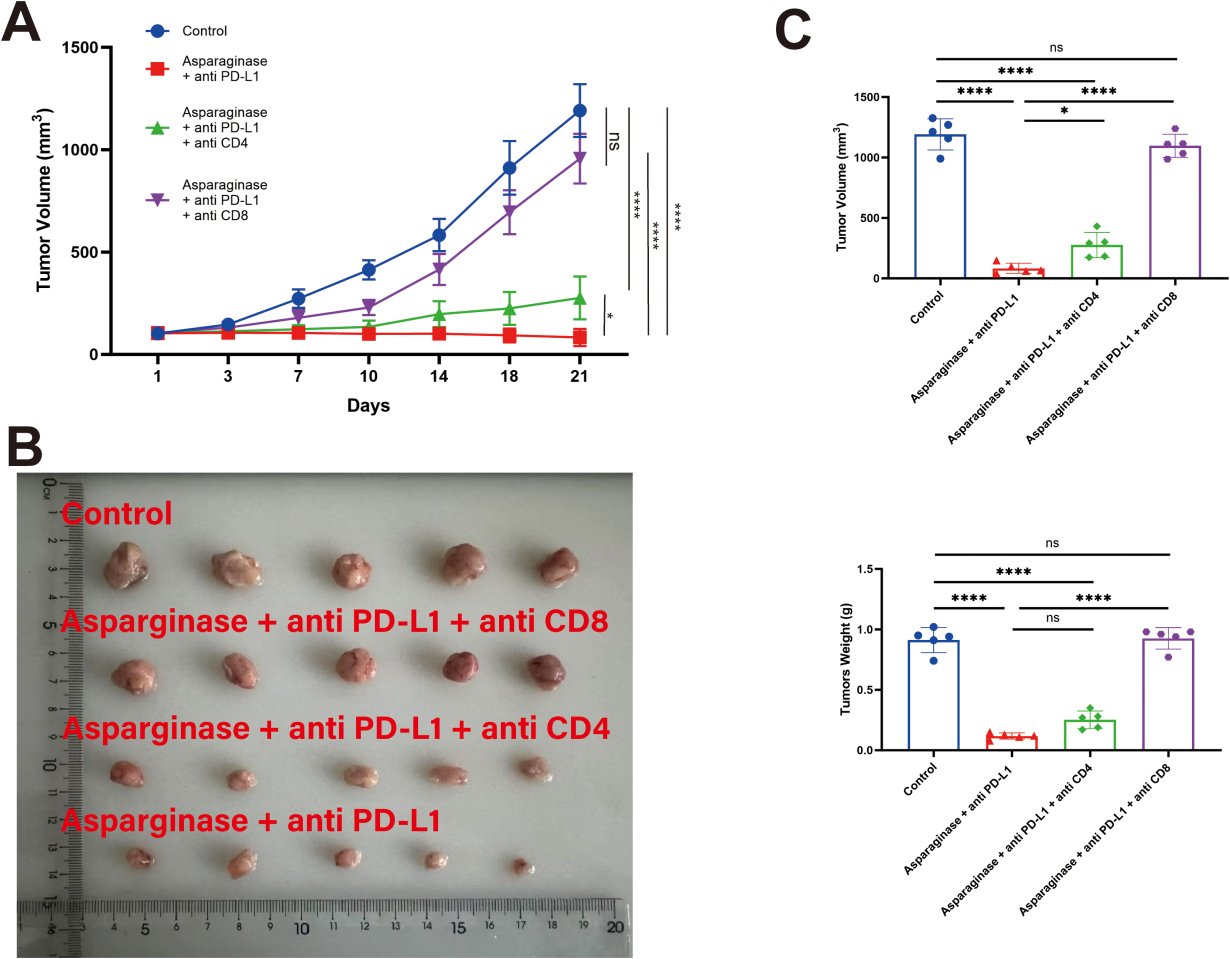
T cell subsets in the antitumor efficacy of co-targeting asparagine and PD-L1. The antitumor effect of the combined therapy was abolished by CD8+ T cell depletion. **(A, B)** Tumor volume was measured twice a week. **(C)** Statistical analysis of tumor weight and volume at the end of the treatment. * indicates p-value < 0.05, ** indicates p-value < 0.01, *** indicates p-value < 0.001 and **** indicates p-value < 0.0001. ns means no significance.

Following specific depletion using anti-CD4 and anti-CD8 antibodies, the anti-tumor effect of the combination therapy was assessed. The tumor growth rate was significantly slower in the Asparaginase + anti-PD-L1 group and the Asparaginase + anti-PD-L1 + anti-CD4 group compared to the Control group (**Figure 7A**). However, depletion of CD8^+^ T cells (Asparaginase + anti-PD-L1 + anti-CD8 group) significantly diminished the anti-tumor efficacy of the combination therapy, resulting in tumor growth rates significantly faster than the Asparaginase + anti-PD-L1 group. Depletion of CD4^+^ T cells did not abolish the efficacy, as the Asparaginase + anti-PD-L1 + anti-CD4 group showed similar efficacy to the Asparaginase + anti-PD-L1 group.

Statistical analysis of final tumor weights (**Figure 7C**) further supported these findings. Control group were 912.00 ± 93.25 mg, Asparaginase + anti-PD-L1 group were 118.00 ± 23.15 mg, Asparaginase + anti-PD-L1 + anti-CD4 group were 252.00 ± 65.23 mg, Asparaginase + anti-PD-L1 + anti-CD8 group were 846.00 ± 79.39 mg. These results collectively demonstrate that the *in vivo* anti-tumor efficacy of the Asparaginase + anti-PD-L1 combination therapy is critically dependent on the presence of CD8^+^ T cells.

## 3 Discussion

This study focused on investigating the impact of targeting asparagine metabolism on the GC immune microenvironment and exploring its potential for combined application with immunotherapy (42–44). Our findings collectively demonstrate that targeting asparagine can significantly influence the composition and function of immune cells within the TME and produce synergistic anti-tumor effects with PD-L1 inhibitors, thereby suggesting a potential strategy for sensitizing GC to immunotherapy.

**Figure 8:**
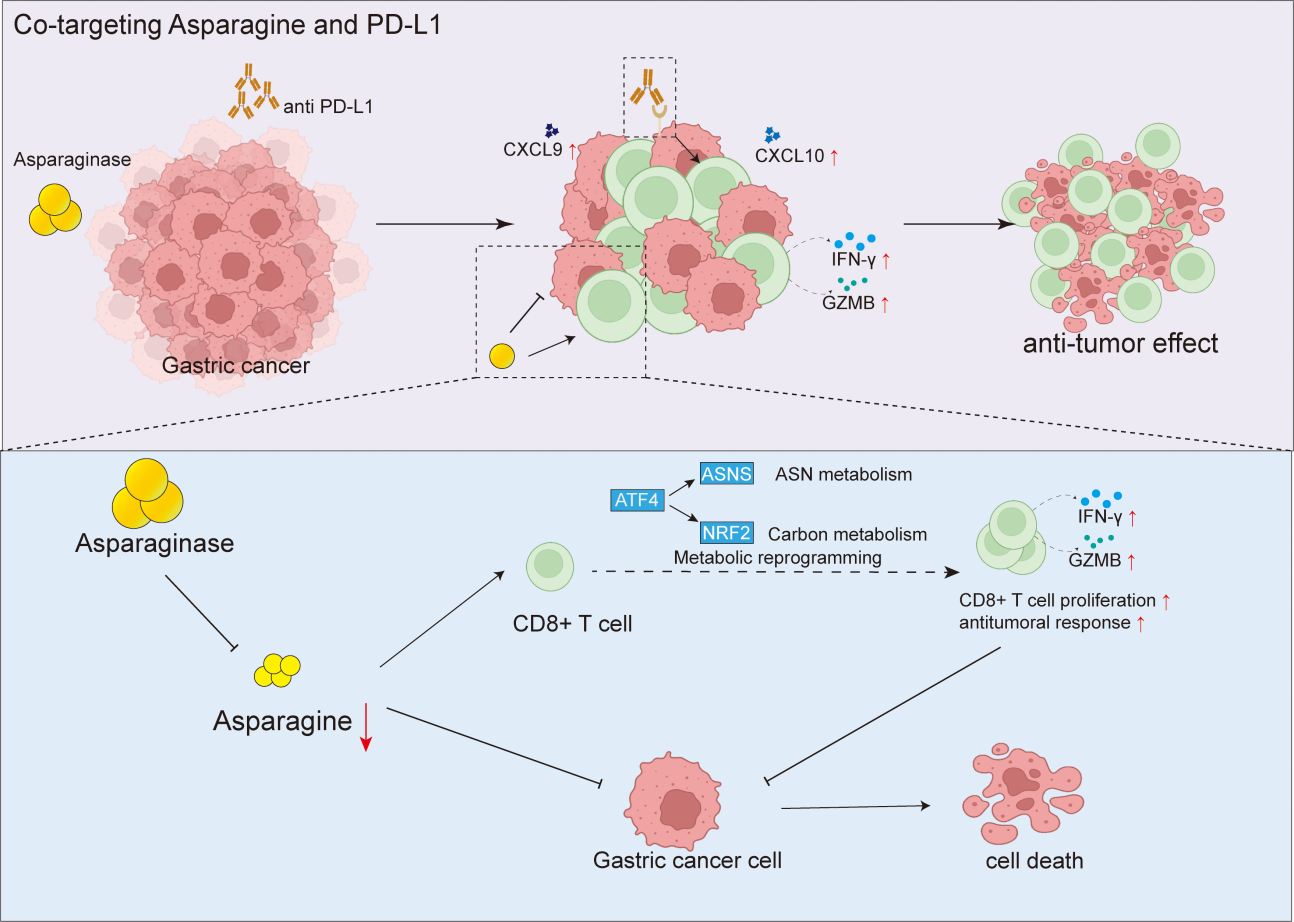
Targeting asparagine potentiates anti-PD-L1 immunotherapy in gastric cancer by enhancing CD8^+^ T cell anti-tumor response.

We first established MFC tumor xenograft models in both C57 mice and nude mice. Asparagine-targeting treatment demonstrated a relatively stronger anti-tumor effect in immunocompetent mice compared to nude mice, suggesting the potential involvement of the immune system in its anti-tumor activity. Further flow cytometry analysis revealed that targeting asparagine significantly increased the proportion of CD8^+^ T cells within tumor tissues. Moreover, it upregulated the expression of their cytotoxicity-related molecules, IFN-γ and GZMB, and promoted the levels of chemokines CXCL9 and CXCL10 known to recruit CD8^+^ T cells. These results indicate that targeting asparagine may enhance anti-tumor immune responses by promoting CD8^+^ T cell infiltration and activation. To further investigate the direct effects of targeting asparagine on CD8^+^ T cells, we conducted *in vitro* experiments. Results showed that asparagine depletion significantly enhanced the proliferative activity of CD8^+^ T cells and promoted their metabolic activity, as evidenced by increased glucose consumption and ATP production. Furthermore, when co-cultured with MFC cells, the secretion of IFN-γ and GZMB by asparagine-targeting treated CD8^+^ T cells were also significantly elevated, further confirming the critical role of asparagine metabolism in regulating CD8^+^ T cell function. These findings are consistent with previous studies reporting a correlation between limited asparagine availability and CD8^+^ T cell proliferative activity (**Figure 8**).

Based on the finding that targeting asparagine can remodel the tumor immune microenvironment, we hypothesized that it might exhibit synergistic effects with immune checkpoint inhibitors. *In vivo* validation demonstrated that combination treatment with asparagine targeting and a PD-L1 inhibitor significantly enhanced the anti-tumor effect against MFC xenografts compared to single-agent therapies. Immunohistochemistry and immunofluorescence analyses further confirmed that the combination therapy more effectively promoted CD8^+^ T cell infiltration into tumor tissue. Treatment with asparagine-targeting and anti-PD-L1 did not result in significant organ toxicity. To clarify the critical role of CD8^+^ T cells in the combination therapy, we inhibited CD4^+^ and CD8^+^ T cell function using antibody blockade experiments. Results indicated that blocking CD8^+^ T cell activity significantly inhibited the anti-tumor effect of the combination treatment, while blocking CD4^+^ T cells had a relatively smaller impact. This result clearly establishes a pivotal role for CD8^+^ T cells in the asparagine-targeting and anti-PD-L1 combination therapy.

Although this study preliminarily validated the effectiveness of combining asparagine targeting with a PD-L1 inhibitor and the critical role of CD8^+^ T cells in mouse models, certain limitations exist. Firstly, the use of the murine GC cell line MFC in xenograft models means the results may not fully represent the complexity of human GC. Secondly, while immune-mediated anti-tumor effects were observed in immunocompetent mice, the specific impact of targeting asparagine and the efficacy of combination therapy within the complex immunosuppressive microenvironment of human GC patients requires further investigation. Future research directions should include validating the efficacy of combining asparagine targeting with immunotherapy in humanized mouse models or patient-derived xenograft (PDX) models. Further exploration of the molecular mechanisms by which targeting asparagine influences the tumor immune microenvironment, such as its regulation of immune cell metabolic pathways and functions, is warranted.

Metabolic reprogramming is recognized as a core hallmark of cancer, enabling tumor cells to meet the biosynthetic and energetic demands required for rapid proliferation. Among various metabolic pathways, the dysregulation of asparagine metabolism plays a pivotal role in the progression of multiple tumors. Although asparaginase, a drug that depletes serum asparagine, has been successfully applied in the clinical treatment of acute lymphoblastic leukemia (ALL), its efficacy in solid tumors such as gastric cancer is limited. This is primarily attributed to the high expression of asparagine synthetase (ASNS) in solid tumor cells, which endows them with a robust capacity for endogenous synthesis to compensate for the deprivation of exogenous asparagine.

This mechanism highlights the importance of ASNS as a potential biomarker for predicting sensitivity to asparagine-targeting therapies. Consequently, future studies must systematically evaluate the baseline expression level of ASNS in gastric cancer cells and its dynamic changes during treatment, which is crucial for stratifying patient populations that may benefit and for elucidating mechanisms of resistance. Building on this, targeting ASNS not only provides a basis for predicting efficacy but also reveals a new therapeutic target. A highly promising therapeutic strategy is to adopt a dual-blockade approach: the combination of asparaginase with an ASNS inhibitor (e.g., ASX-173) to simultaneously target both exogenous uptake and endogenous synthesis pathways. This combination therapy is expected to achieve a more thorough disruption of asparagine metabolism, thereby producing a synergistic and potent anti-tumor effect.

Specifically, we have now included several key future directions, Screening for biomarkers to identify subgroups of gastric cancer patients who may benefit from asparagine-targeted therapy. Exploring the potential of combining asparagine-targeted therapy with other immunotherapies, such as CAR-T cells or tumor vaccines. Elucidating the specific mechanisms by which asparagine affects other immune cells, including NK cells and macrophages. We have emphasized that this study provides a new target and theoretical basis for “metabolic-immunotherapy” combination strategies. We hope this will offer inspiration and direction for future researchers in developing more effective immunotherapy strategies for gastric cancer. Finally, conducting clinical trials to evaluate the safety and efficacy of combining asparagine targeting with immune checkpoint inhibitors in patients with advanced GC is a crucial next step for translating this strategy into clinical practice.

## 4 Methods

### 4.1 Reagents and antibodies

Asparaginase (H20153215) were purchased from Jiangsu Hengrui Pharmaceuticals Co., Ltd. InVivoMAb anti-mouse PD-L1 (#BE0101) were purchased from Bioxcell.

### 4.2 Asparagine levels analysis

Asparagine concentration was measured using the Asparagine Kits (JONLNBio, JL-T1108) according to the manufacturer’s instructions.

### 4.3 Tumor cell line culture

MFC was acquired from the Type Culture Collection of the Chinese Academy of Sciences (Shanghai, China). MFC was cultured in DMEM Medium supplemented with 10% FBS, 100 U/mL penicillin and 100 μg/mL streptomycin. Cells were maintained in a humidified incubator at 37 °C with 5% CO_2_.

### 4.4 *In vivo* experiments

All mice were housed in a specific pathogen‐free facility in the Laboratory Animal Center of Changhai hospital, with 5 mice per cage, according to the guidelines of the National Academy of Sciences and the National Institutes of Health. Permission of animal experiments was obtained from the Committee on Ethics of Medicine, Naval Medical University, PLA (2024-067).

To establish a subcutaneous tumor model, Mouse MFC GC cells (3×10^6^) suspended in 100 µL PBS) were subcutaneously implanted into the flank of male C57BL/6 mice. The mice were 6–8 weeks old and weighed between 19–21 g at the initiation of the study. Tumor growth was monitored regularly, and tumor volumes were determined by caliper measurements of the length and width of the tumors. The volume was calculated using the standard formula: (length × width²)/2.

Following tumor establishment, mice were randomized into treatment groups. Therapeutic interventions consisted of intraperitoneal (i.p.) administration of Asparaginase at a dose of 2 IU/g body weight, an anti-PD-L1 antibody at 200 µg per mouse, or a combination regimen of Asparaginase (2 IU/g) and anti-PD-L1 (200 µg). All therapeutic agents were delivered via intraperitoneal injection.

### 4.5 Mouse CD8^+^ T cell isolation and culture

CD8+ T cells were isolated from C57BL/6 mice aged 6–8 weeks. Single-cell suspensions were prepared from spleens by mechanical disruption through a 70 μm cell strainer. Red blood cells were lysed using a red blood cell lysis buffer. CD8+ T cells were then positively selected using a CD8a+ T Cell Isolation Kit according to the manufacturer’s instructions. The purity of the isolated CD8+ T cells was typically > 90% as determined by flow cytometric analysis.

### 4.6 ATP levels analysis

ATP levels were measured using the ATP Assay Kit (Meilunbio, MA0440-1) according to the manufacturer’s instructions.

### 4.7 Glucose levels analysis

Glucose concentration was measured using the Glucose Assay Kit (Yeasen, 60408ES60) according to the manufacturer’s instructions.

### 4.8 Western blot

All antibodies used for Western blot were purchased from Thermofisher. β-actin antibody (Thermofisher, MA1-140), ASNS antibody (Thermofisher, MA1-703164), ATF4 antibody (Thermofisher, MA5-32364), NRF2 antibody (Thermofisher, PA5-27882).

### 4.9 ELISA kits

All ELISA kits were purchased from Abclonal. The concentrations of mouse IFN-gamma, Granzyme B, CXCL9, and CXCL10 were determined using specific ELISA kits from Mouse IFN-gamma ELISA Kit (Abclonal, RK00019), Mouse Granzymes B ELISA Kit (Abclonal, RK00370), Mouse CXCL 9 ELISA Kit (Abclonal, RK03026), Mouse CXCL10 ELISA Kit (Abclonal, RK00056).

### 4.9 Flow cytometric

All antibodies used for Flow cytometric were purchased from BioLegend. Single-cell suspensions were prepared from samples, and red blood cells were removed by lysis. Non-specific binding was blocked by pre-incubation with blocking antibody. Subsequently, cells were stained with fluorophore-conjugated antibodies, including APC anti-mouse CD4 Antibody and PerCP anti-mouse CD8 Antibody, for 30 minutes in the dark. Following incubation, cells were washed with flow cytometry buffer. Data acquisition was performed using a BD flow cytometer (specify model if known), and analysis was conducted using FlowJo software (version 10.0).

### 4.10 Histology and immunohistochemistry

Tumor tissues were then fixed, embedded in paraffin, and sectioned. A citrate antigen retrieval solution (pH 6.0) was employed to restore the antigen, while endogenous pe roxidase was inhibited by incubating the samples in a 3% hydrogen peroxide solution for 25 minutes at room temperature, shielded from light. To block the samples, a 3% BSA solution was applied and incubated for 30 minutes at room temperature. Primary antibody, HRP-labeled secondary antibody were added sequentially and incubated for 50 minutes, redundant antibody was washed away by PBS (PH = 7.4) for three times and then added diaminobenzidine chromogenic solution. Microscope was utilized to control time of chromogenic development. The nuclei were re-stained with hematoxylin, after dehydrating, the slices were sealed by neutral dendrimer. Microscope was used to visualize and analyze the results.

### 4.11 T cells depletion

All antibodies used for T-cell depletion were purchased from bioxcell. Mice were injected intraperitoneally with an anti-CD4 antibody (200μg, clone GK1.5, BE0003-1), anti-CD8 antibody (200μg, clone 2.43, BE0061), and Isotype control (IgG2b, 200μg, clone LTF-2, BE0085) on days 1, 4, 7 and 10 respectively.

### 4.12 Statistical analysis

All statistical analyses were performed using GraphPad Prism software (Version 9.0). Data are presented as the mean ± standard error of the mean (SEM). For *in vivo* experiments involving multiple groups and two independent variables, data were analyzed using a two-way analysis of variance (ANOVA) followed by Tukey’s multiple comparisons test to identify specific between-group differences. For comparisons between two independent groups, an unpaired, two-tailed Student’s t-test was used. For comparisons of paired samples from the same subject, a paired, two-tailed Student’s t-test was employed. A P-value less than 0.05 was considered statistically significant. Significance levels are denoted in the figures as follows: *P < 0.05, **P < 0.01, and ***P < 0.001. ‘ns’ indicates a non-significant difference.

## Data Availability

The datasets presented in this study can be found in online repositories. The names of the repository/repositories and accession number(s) can be found in the article/**Supplementary Material**.

## Supplementary material

Supplementary Figure 1
**(A)** The viability of MFC cells treated with ASN, Asparaginase, or Asparaginase plus ASN for 48 h was measured using a CCK-8 assay (n=6). **(B, C)** Western blot analysis of the protein expression levels of Asparagine Synthetase (ASNS) and the autophagy-related protein LC3 in MFC cells following the same treatments. β-actin was used as a loading control. Data are presented as mean ± SD. **P < 0.01, ****P < 0.0001 compared to the control group.
