# Supplementary figures and images for: Targeting asparagine potentiates anti-PD-L1 immunotherapy in gastric cancer by enhancing CD8+ T cell anti-tumor response
Source: Front Immunol. 2025 Oct 28;16:1626581. doi: 10.3389/fimmu.2025.1626581 (PMC12603620; doi:10.3389/fimmu.2025.1626581)

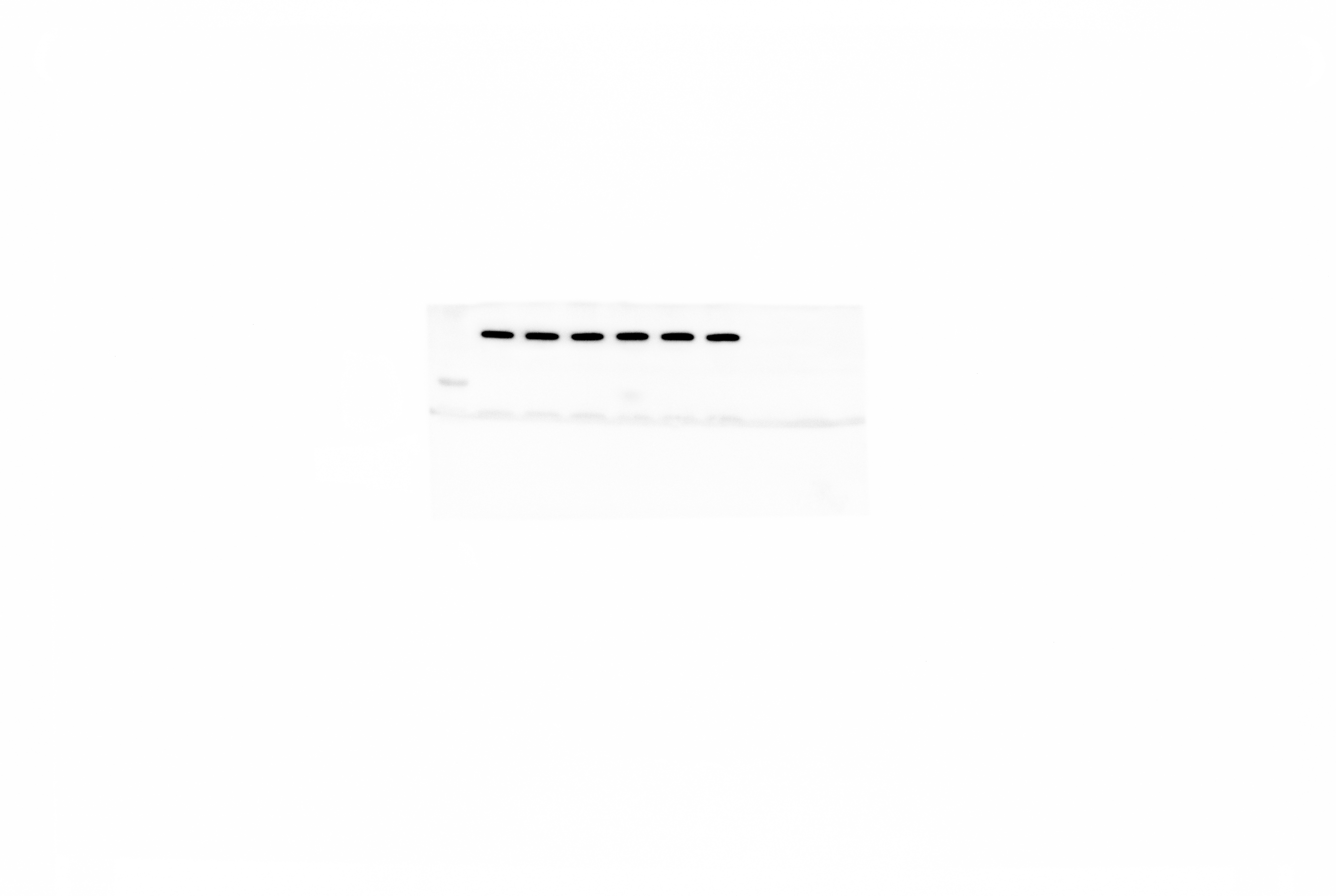

Supplement: Supplementary Figure 1 — (A) The viability of MFC cells treated with ASN, Asparaginase, or Asparaginase plus ASN for 48 h was measured using a CCK-8 assay (n=6). (B, C) Western blot analysis of the protein expression levels of Asparagine Synthetase (ASNS) and the autophagy-related protein LC3 in MFC cells following the same treatments. β-actin was used as a loading control. Data are presented as mean ± SD. **P < 0.01, ****P < 0.0001 compared to the control group. [file Image1.tiff]

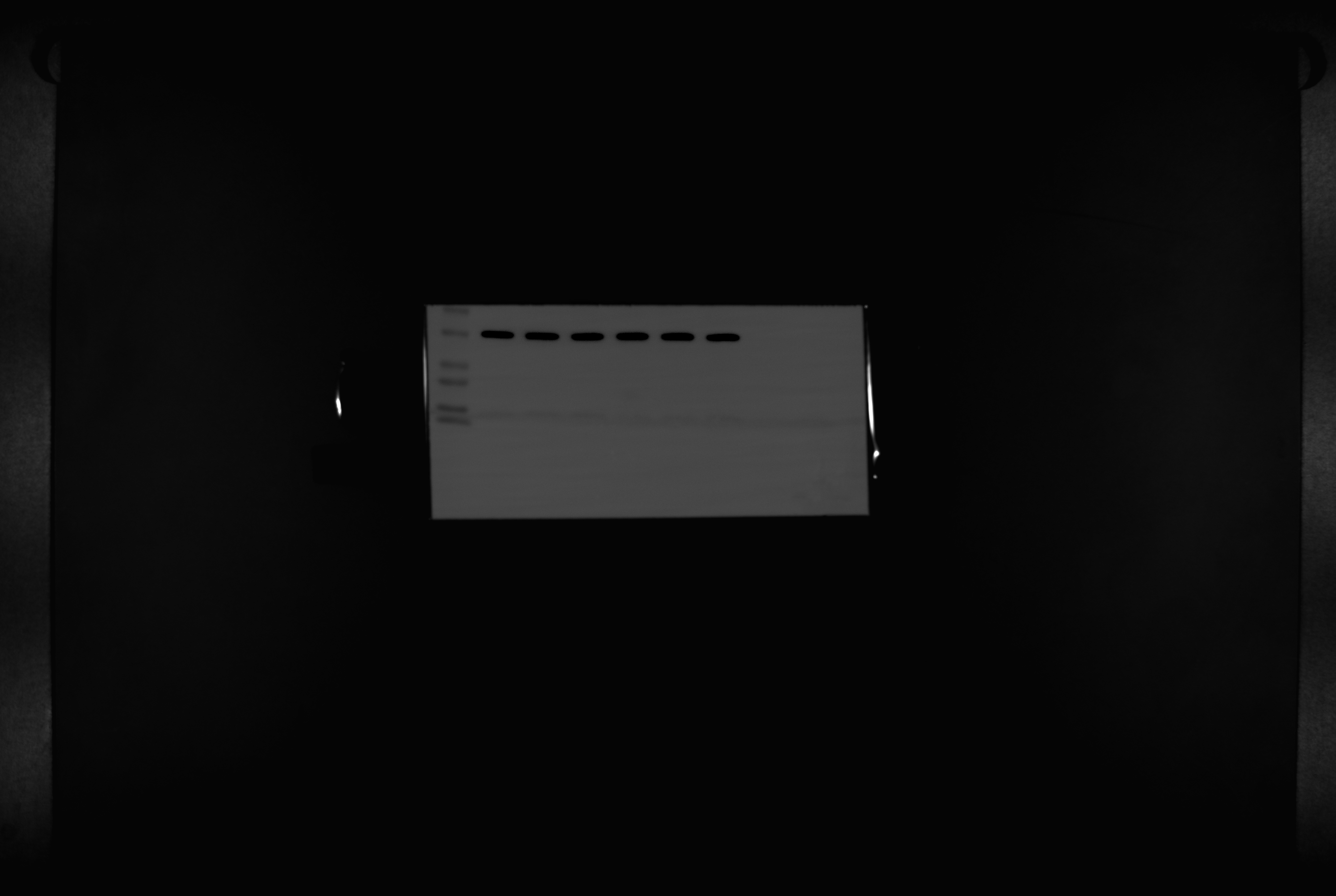

Supplement: Supplementary file 2 [file Image2.tiff]

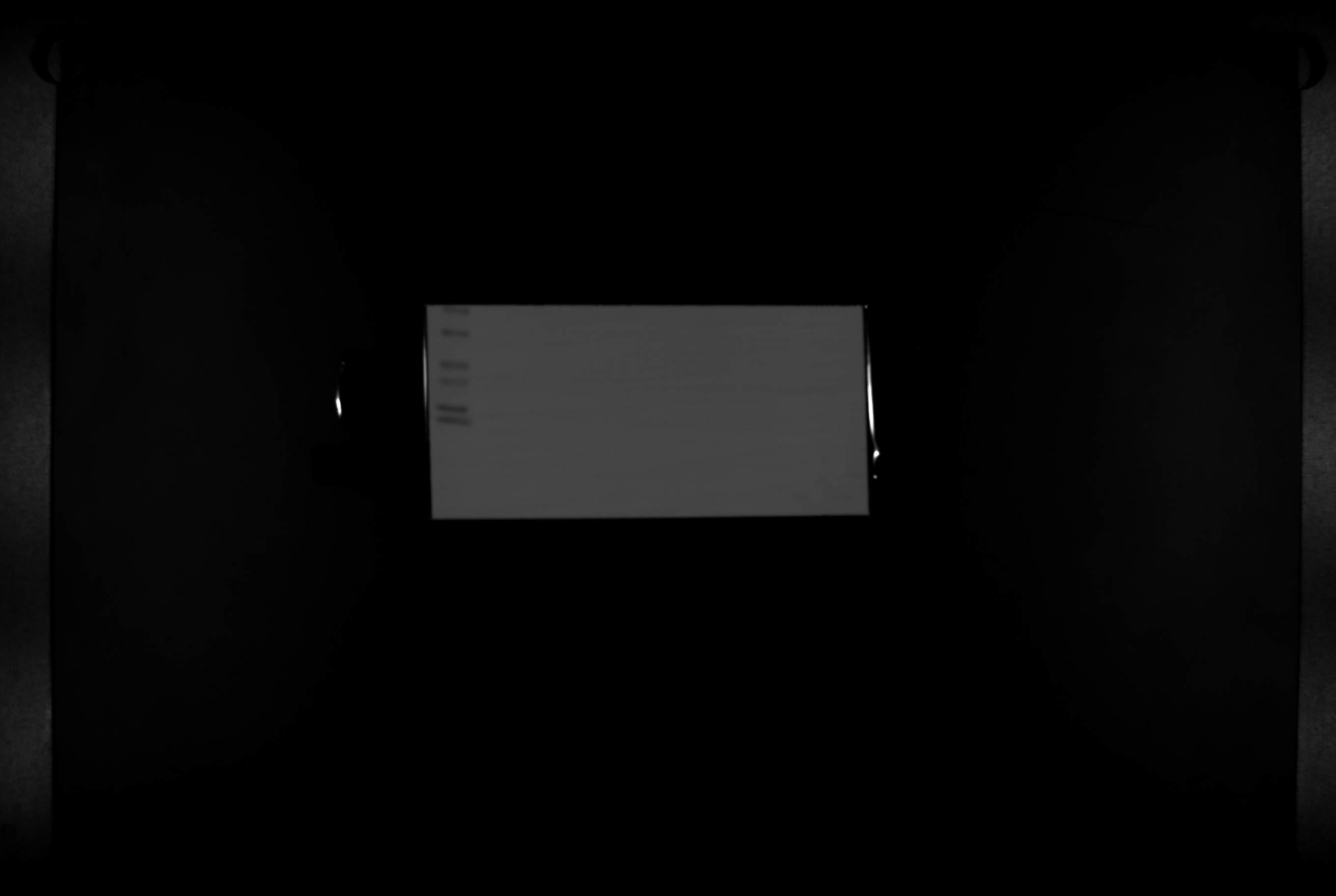

Supplement: Supplementary file 3 [file Image3.tiff]

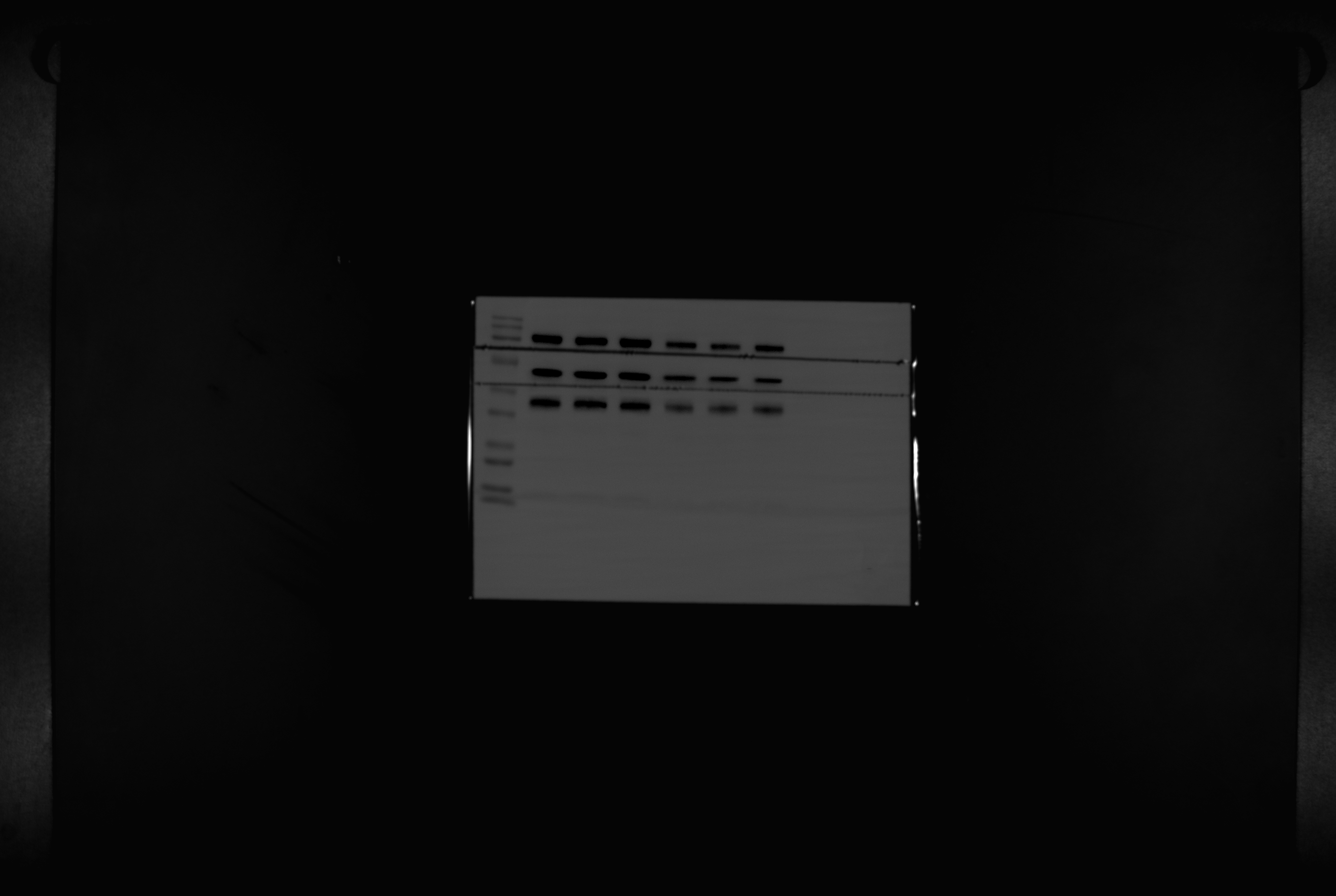

Supplement: Supplementary file 4 [file Image4.tiff]

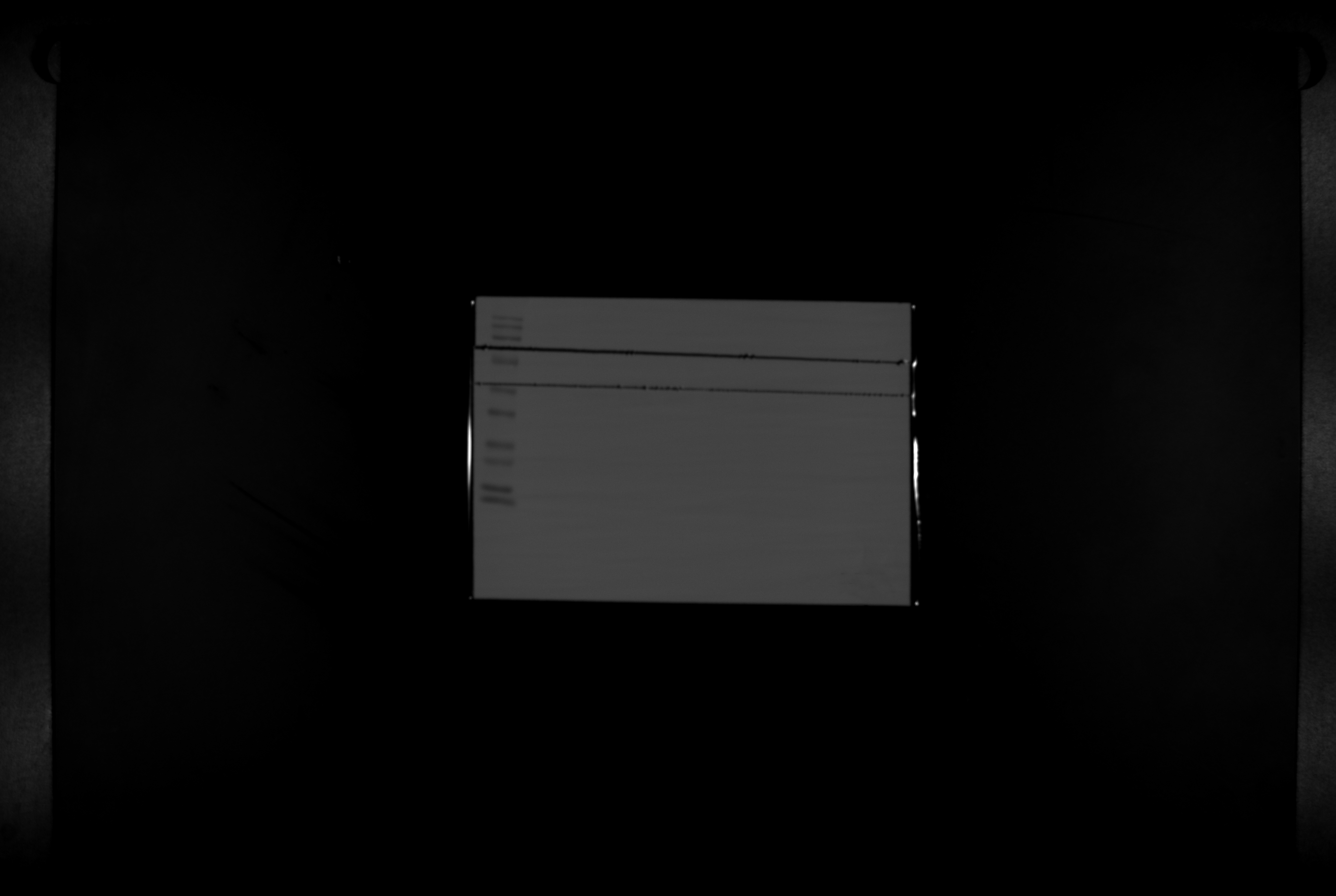

Supplement: Supplementary file 5 [file Image5.tiff]

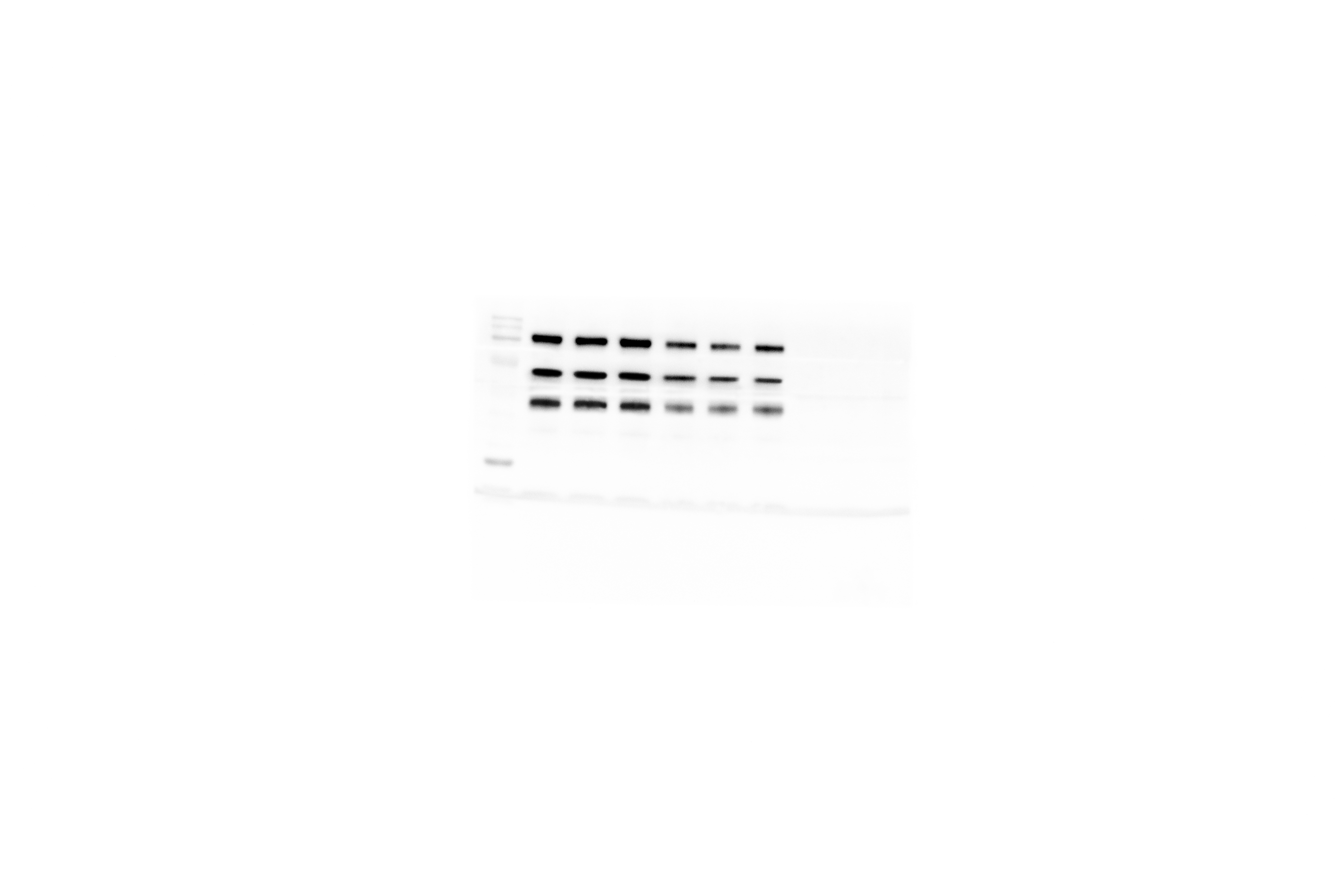

Supplement: Supplementary file 6 [file Image6.tiff]

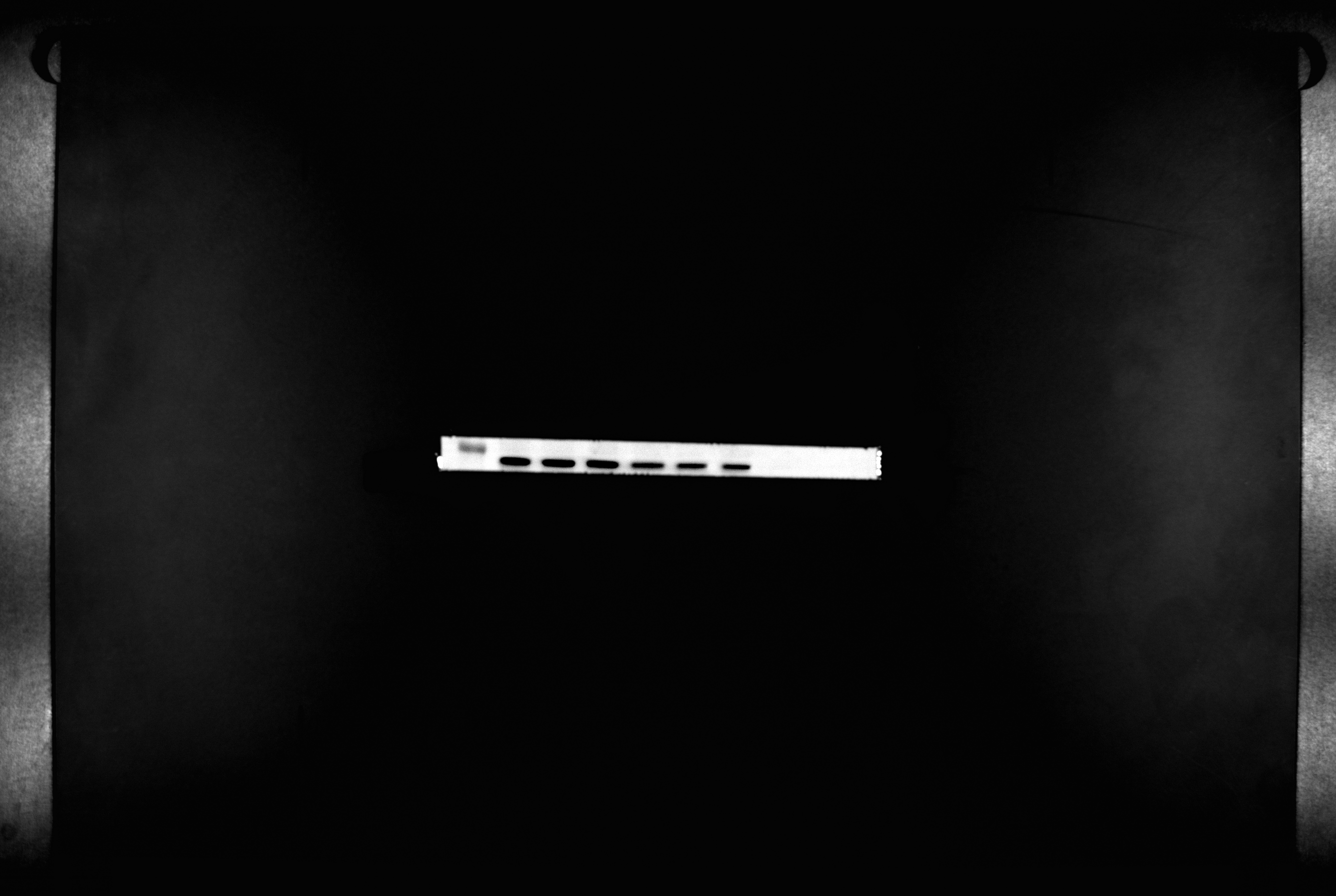

Supplement: Supplementary file 7 [file Image7.tiff]

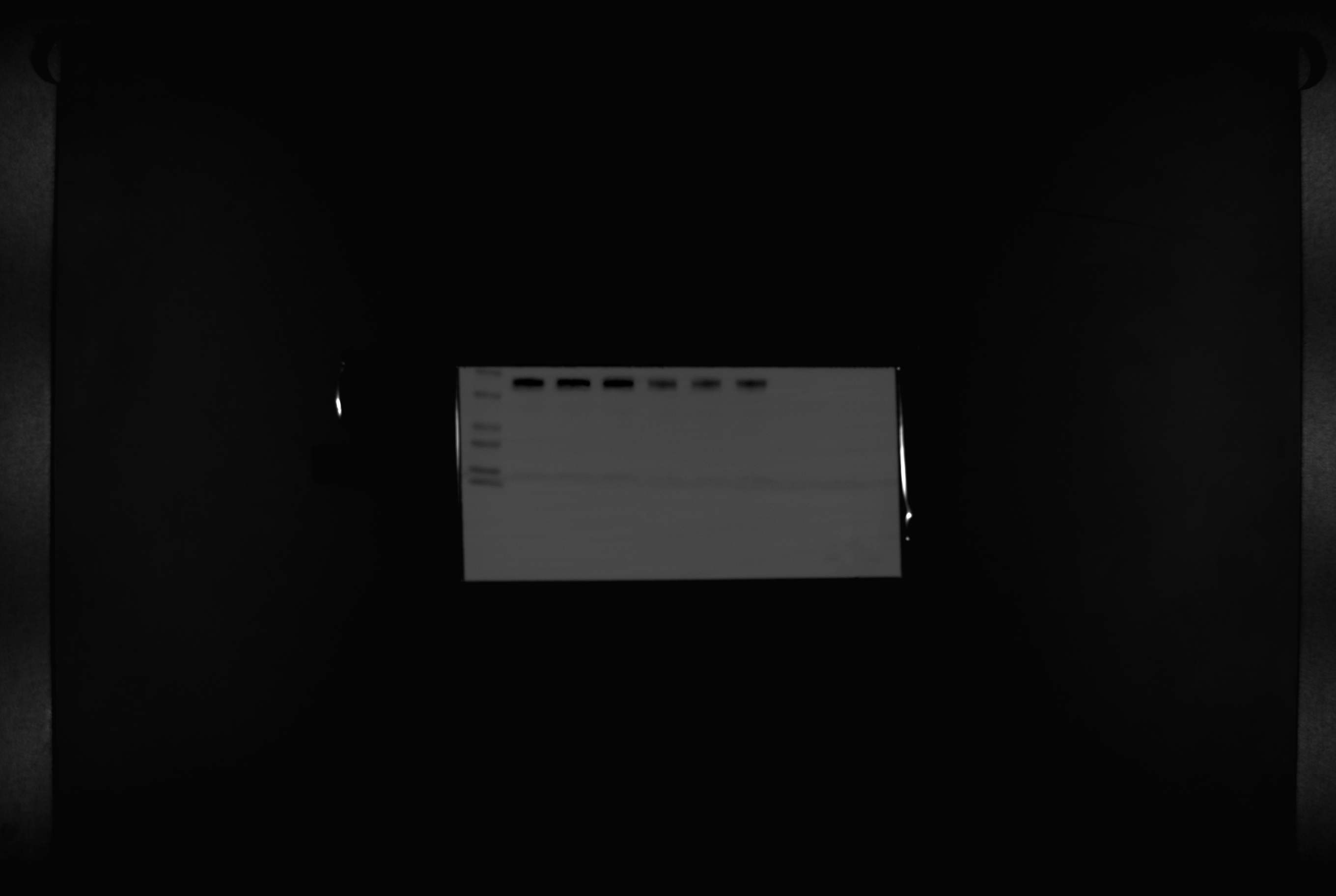

Supplement: Supplementary file 8 [file Image8.tiff]

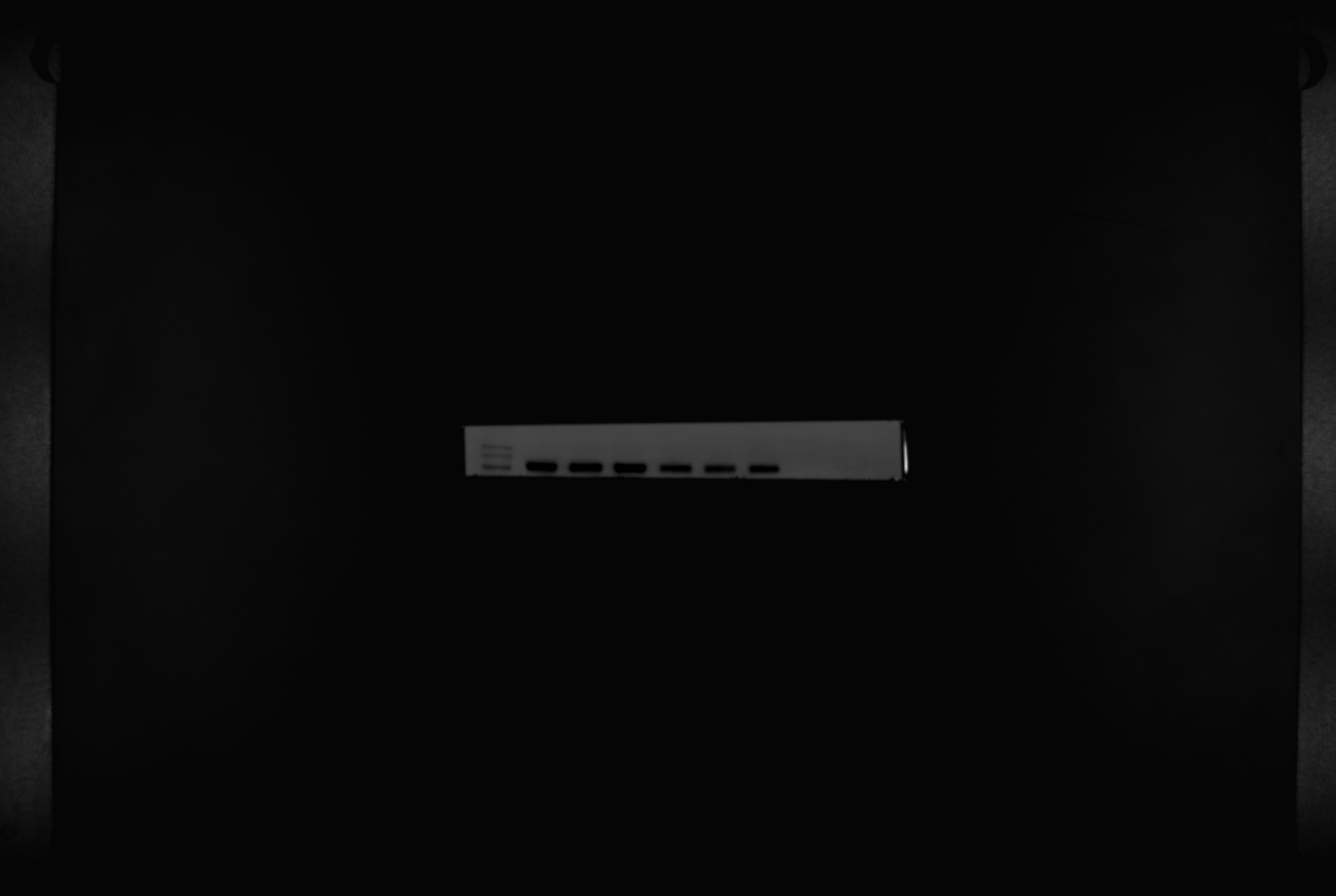

Supplement: Supplementary file 9 [file Image9.tiff]

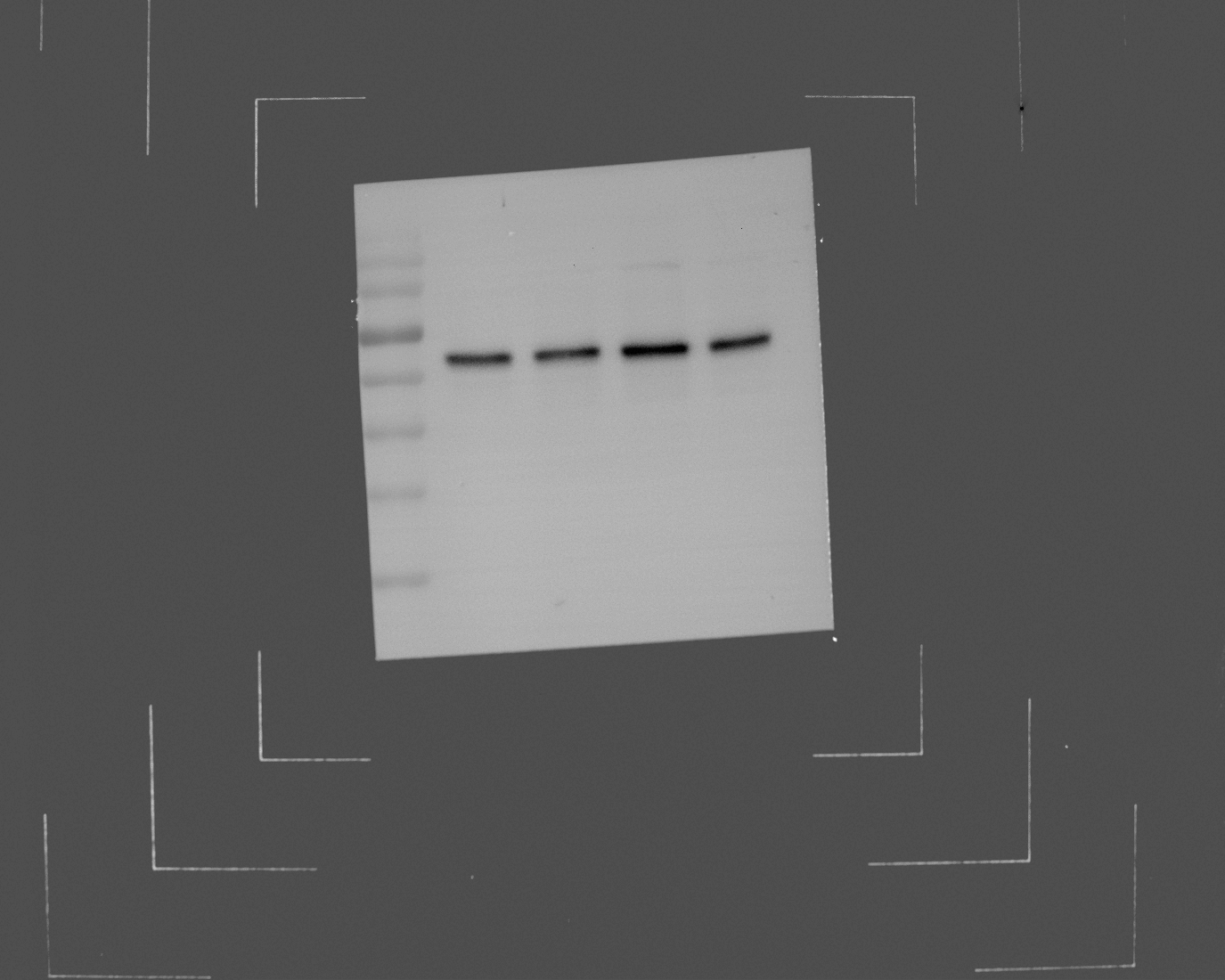

Supplement: Supplementary file 10 [file Image10.tif]

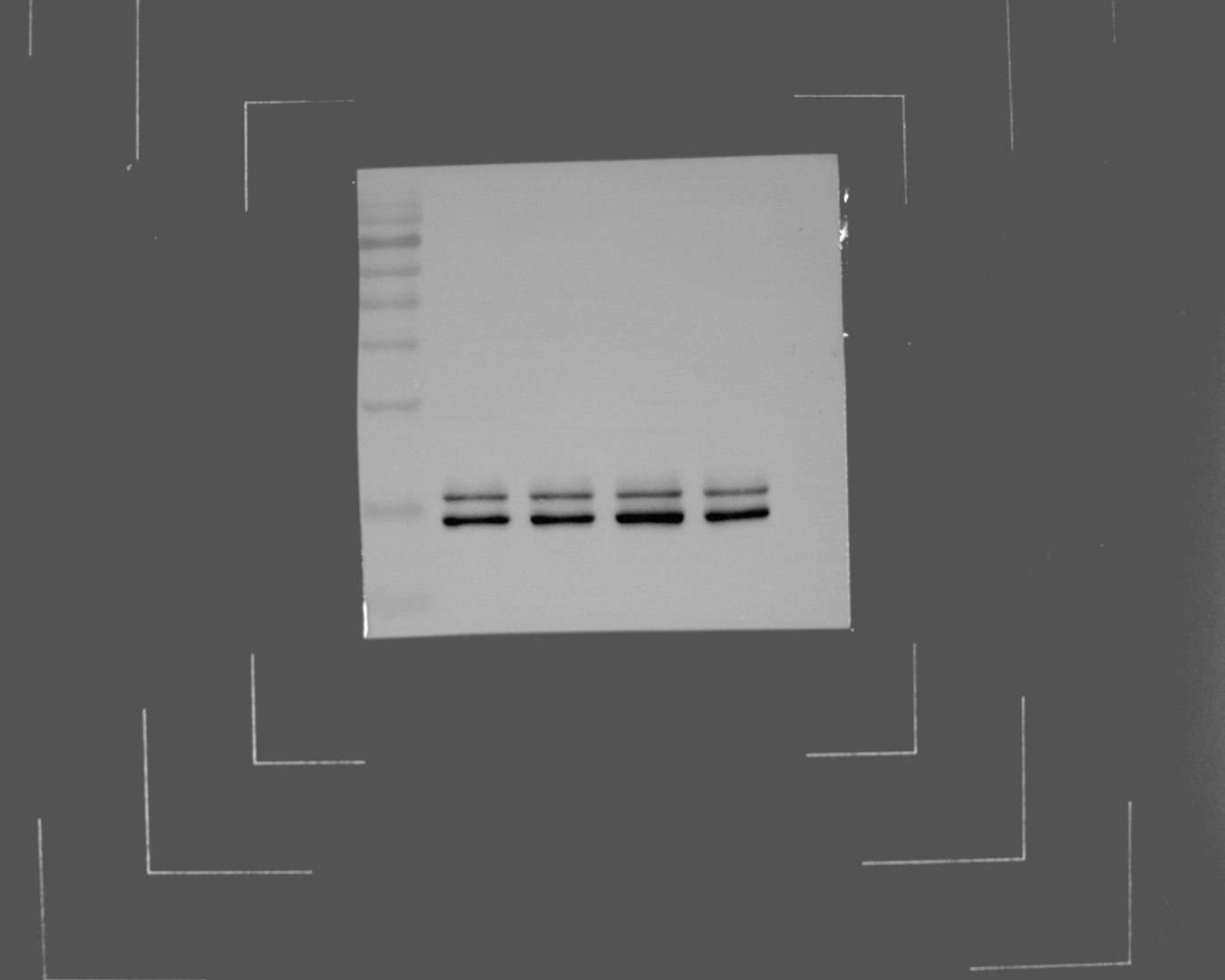

Supplement: Supplementary file 11 [file Image11.tif]

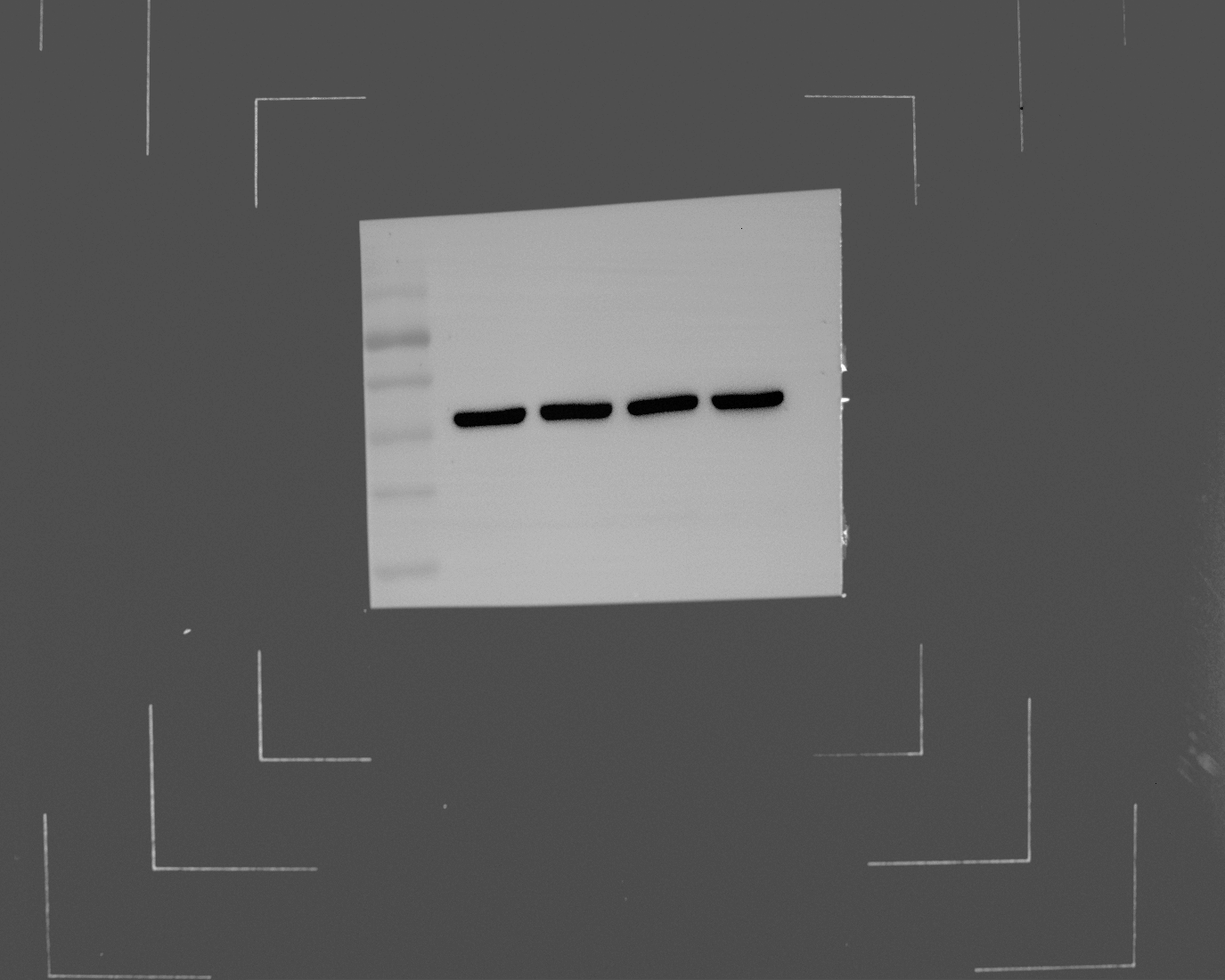

Supplement: Supplementary file 12 [file Image12.tif]

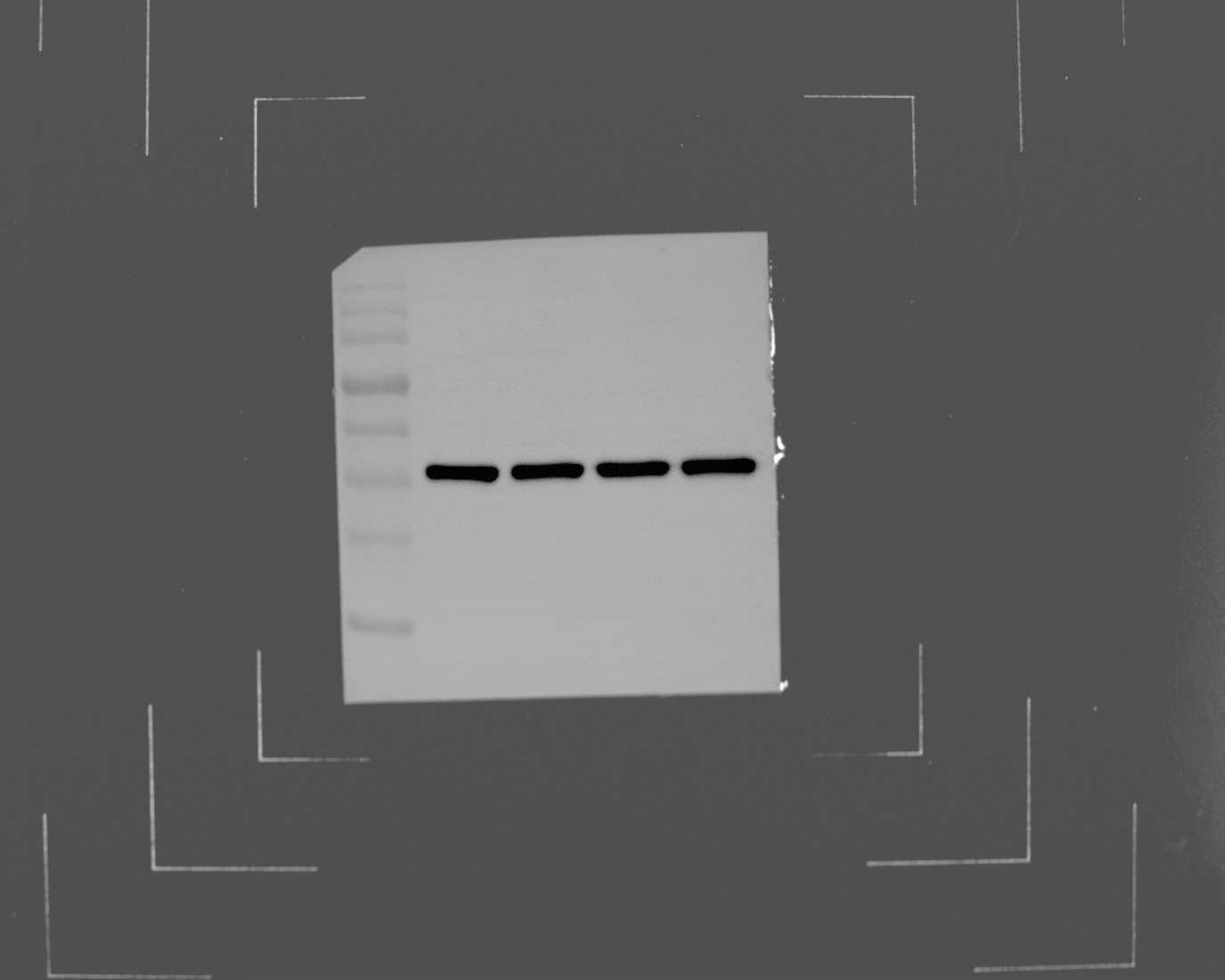

Supplement: Supplementary file 13 [file Image13.tif]

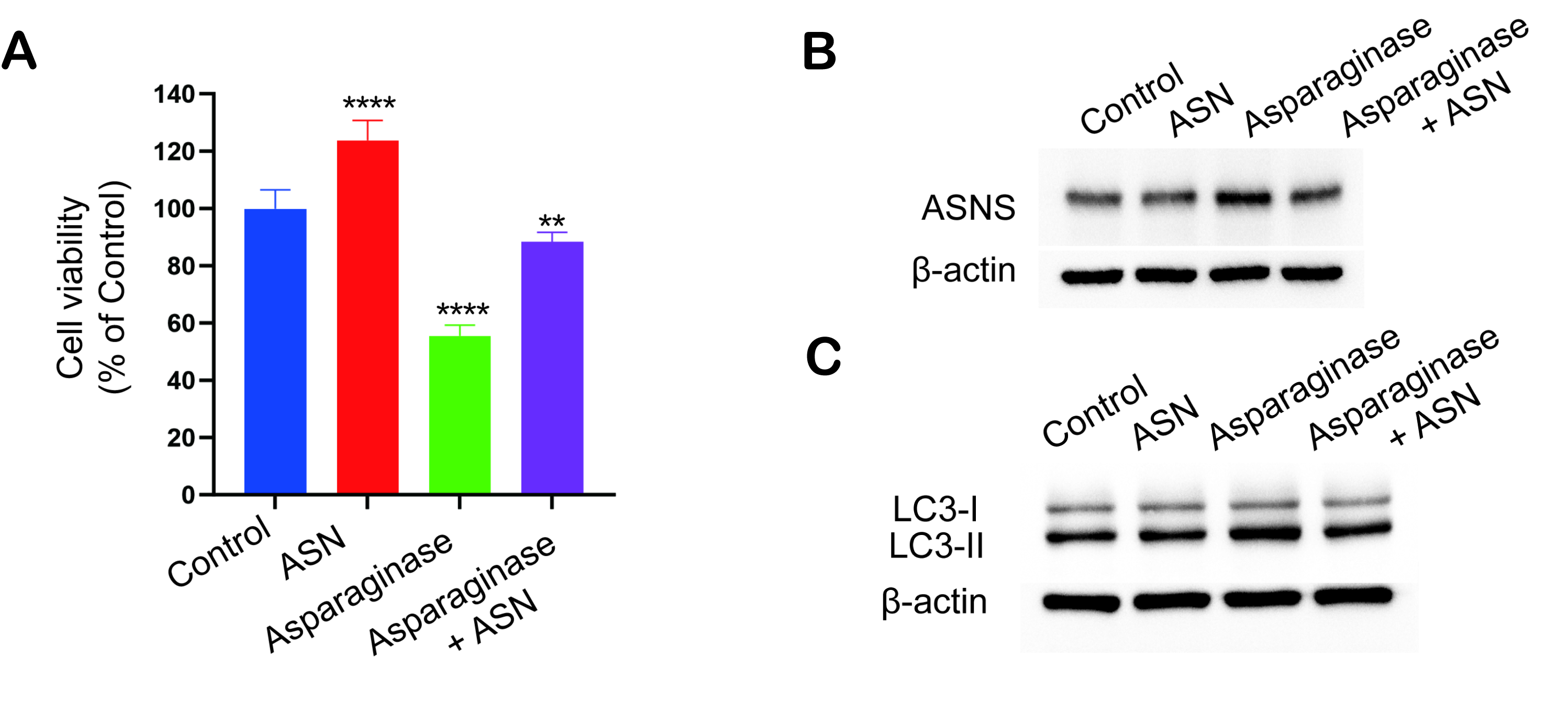

Supplement: Supplementary file 14 [file Image14.tif]
